# Network Pharmacology-Based Study on the Mechanism of Bushen-Jianpi Decoction in Liver Cancer Treatment

**DOI:** 10.1155/2019/3242989

**Published:** 2019-03-19

**Authors:** Rong Wu, Xiao-Yan Li, Wen-Hai Wang, Fei-Fei Cai, Xiao-Le Chen, Meng-Die Yang, Qiu-Sha Pan, Qi-Long Chen, Rong-Yao Zhou, Shi-Bing Su

**Affiliations:** ^1^Research Center for Traditional Chinese Medicine Complexity System, Institute of Interdisciplinary Integrative Medicine Research, Shanghai University of Traditional Chinese Medicine, China; ^2^Shanghai Baoshan Hospital of Integrated Traditional Chinese Medicine and Western Medicine, Shanghai University of Traditional Chinese Medicine, Shanghai 201999, China; ^3^Department of Medical Oncology, Shuguang Hospital, Shanghai University of Traditional Chinese Medicine, Shanghai 201203, China

## Abstract

To investigate the mechanism of a Bushen-Jianpi decoction (BSJPD) in liver cancer (LC) treatment, we analyzed clinical therapy data, conducted network pharmacology analysis, and performed pharmacological experimental verification* in vitro* and* in vivo*. The univariate analysis of clinical therapy showed that the BSJPD was protective factor (*p* < 0.05). The network pharmacology analysis showed that 9 compounds were important nodes of BSJPD-LC therapy network. In experimental verification, the rate of apoptosis increased in the liver tumors of mice treated with the BSJPD (*p* < 0.05); drug serum with 20 % BSJPD inhibited cell viability (*p* < 0.05) and reduced the expression of PI3K, the Bcl-xL/BAD ratio, and the levels of p53 and p-Akt in HepG2 cells. Moreover, licochalcone A, alisol B, and hederagenin inhibited cell viability (*p* < 0.05), induced cell apoptosis (*p* < 0.01), reduced p-Akt levels, and increased cleaved-CASP3 (*p* < 0.05) and p53 expression levels in HepG2 cells. These data suggest that the BSJPD prolongs the survival of LC patients and induces apoptosis and that it may be associated with the regulation of PI3K, Akt, p53, CASP3, and Bcl-xL/BAD expression.

## 1. Introduction

Liver cancer (LC), a frequently occurring cancer, has become the second leading cause of cancer mortality [1–3]. LC is the sixth most common cancer, and most patients are diagnosed and treated at the clinical III or IV stage [2]. Thus, few treatments can be provided, apart from sorafenib and transcatheter arterial chemoembolization (TACE) [4, 5]. In recent years, the survival time has still been terribly low [6].

In the clinic, traditional Chinese medicine (TCM) plays a significant role in LC treatments. There were many studies on the treatment of LC by TCM and monomers TCM agents [7, 8]. Bushen-Jianpi decoction (BSJPD) is a combination of Liu-Wei-Di-Huang decoction (LWDHD) and Si-Jun-Zi decoction (SJZD). Previous pharmacological studies have reported that LWDHD and SJZD are effective in treating LC, type 2 diabetes [9], inflammation, and oxidative stress [10] as well as maintaining intestinal homeostasis [11]. As a combination of LWDHD and SJZD, BSJPD is therapeutically used in LC [12]. Because it is not easy to analyze the compounds in BSJPD by traditional pharmacological evaluations, the mechanism of action of BSJPD in LC is still unclear.

As a new field in modern TCM pharmacological studies, network pharmacology can be used to explore the mechanisms of action of TCMs as disease treatments using many existing databases, pathway analysis, and network analysis [2, 13]. Network pharmacology is focused on the compounds and targets in the interactome. It is suitable for exploring the mechanisms of action of TCMs and their synergistic effects in cancer therapy [7]. The drug-target network, a vital part of network pharmacology, plays a role in interpreting the mechanisms of complex compounds. Therefore, we conducted the current study to demonstrate the benefits of BSJPD treatment on survival and to clarify the effective mechanisms of BSJPD on LC by survival analyses, network analysis of compound-target pathways, and* in vitro* pharmacological experimental verification.

## 2. Materials and Methods

### 2.1. Reagents

Quercetin (HPLC ≥ 98 %), kaempferol (HPLC ≥ 98 %), hederagenin (HPLC ≥ 98 %), *β*-sitosterol (HPLC ≥ 98 %), stigmasterol (HPLC ≥ 98 %), formononetin (HPLC ≥ 98 %), isorhamnetin (HPLC ≥ 98 %), alisol B (HPLC ≥ 98 %), licochalcone A (HPLC ≥ 98 %), morroniside (HPLC ≥ 98 %), loganin (HPLC ≥ 98 %), and paeonol (HPLC ≥ 98 %) were purchased from the Standardization Research Center of TCM (Shanghai, China). The concentrations of DMSO in this study were < 0.1 %. The compounds are stable under the experimental conditions. All chemical structures are shown in Supplementary Figure 1.

5-Fluorouracil (lot: 100201) was obtained from Shanghai XuDong Haipu Pharmaceutical Co., Ltd.

Tetrazolium compound 3-(4,5-dimenthyl-2-yl)-5-(3-carboxymethoxyphenyl)-2-(4sulfophenyl)-2H-tetrazolium MTS (lot: 313103) was obtained from Promega (USA). IFN-*γ* (lot: 53680), IL-10 (lot:51324) and IL-12-P40 (lot: 53103) assay kits were obtained from BD Biosciences Pharmingen (USA). TNF-*α* (lot: 96-300-01A-50) was obtained from Peprotech (USA). Muse™ Annexin V Dead cell kits (lot: 3026089) were purchased from EMD Millipore (USA). Antibodies against GAPDH (lot: 5174, 2), ACTB (lot: 3700, 19), cleaved-caspase-3 (lot: 9661, 25), caspase-3 (lot: 9662, 17), BAD (lot: 9268, 4), Bcl-xL (lot: 2764, 9), p-mTOR (lot: 5536, 7), mTOR (lot: 2983, 6), PI3K (lot: 4263, 5), p-Akt (lot: 4060, 23), Akt (lot: 9272, 23), and p53 (lot: 2524, 26) were purchased from Cell Signaling Technology (USA). The HepG2 and H22 cells were obtained from the Type Culture Collection of the Chinese Academy of Sciences (Shanghai, China).

### 2.2. BSJPD Preparation

The Bushen-Jianpi decoction (BSJPD) contained 15 g* Rehmannia glutinosa* (Gaertn.) DC., 9 g of* Cornus officinalis* Siebold & Zucc., 9 g* Dioscorea oppositifolia* L., 9 g* Panax ginseng* C.A. Mey., 9 g* Atractylodes macrocephala* Koidz., 15 g* Poria cocos* (Schw.) Wolf., 9 g* Alisma plantago-aquatica* subsp. orientale (Sam.) Sam., 9 g* Paeonia suffruticosa* Andrews, and 6 g* Glycyrrhiza uralensis* Fisch. ex DC. All herbs were fully validated using mpns.kew.org. These herbs were purchased from Shu Guang Hospital, Shanghai University of TCM. All herbs were soaked in 2 l water for 30 min, boiled for 30 min, and filtered three times. Finally, a concentration of 5.7 g drug/ml was made.

HPLC-MS MRM chromatograms of the BSJPD extracts and the BSJPD-medicated serum samples are shown in Supplementary Figure 2. Morroniside, loganin, and paeonol were used as the quality control indicators for the BSJPD extracts and the BSJPD-medicated serum samples.

### 2.3. Animals and BSJPD-Medicated Serum Preparation

Sprague-Dawley (SD) rats were assigned to the BSJPD and control groups according to random number assignments. Rats in the BSJPD group received intragastric administration of BSJPD (57 g/kg) twice per day, while the control rats received the same volume of water. On the third day, the rats were anesthetized. The blood from the abdominal aorta was centrifuged into serum and preserved at -80°C. The preparation of the BSJPD-medicated serum was similar to that described by Meng et al. [14]. The SD rats were obtained from Shanghai Slaccas Experimental Animal Co. Ltd., Shanghai, China.

Male Kun-ming (KM) mice were assigned to three groups (n=12). All mice received a subcutaneous injection of 0.2 mL H22 cells (1×10^10^ L^−1^) in the armpit. The treatment started on the sixth day after injection. The mice in the model group were injected with 5-FU (100 mg/kg/48 h, i.p.) seven times. The mice in the BSJPD group were intragastric administered BSJPD (5.7 g/kg) once per day. The model mice were intragastrically administered distilled water (the solvent for BSJPD). The mice were euthanized 15 days after the subcutaneous injection. Body weight and tumor weight were recorded. The apparent diffusion coefficient was measured by Philips Intera Achieva 1.5 T magnetic resonance equipment (Shu Guang Hospital, Shanghai University of TCM). Serum was collected and preserved at -80°C. The BSJPD extraction method and the dosage were performed according to our previous study [12]. The KM mice were obtained from the Shanghai Research Center of the Southern Model Organisms, Shanghai, China.

This protocol was approved by the Animal Care and Use Committee of the Shanghai University of Traditional Chinese Medicine (SZY201709014) in accordance with the recommendations of the Care and Use of Laboratory Animals published by the US National Institutes of Health (NIH Publication No. 85-23, revised 1996).

### 2.4. Patient Characteristics and Treatment

Seventy-six patients with hepatic carcinoma (clinical stage I-IV) were studied between July 2006 and November 2017 at the Shanghai University of TCM Shu Guang Hospital. Patients in the BSJPD group were treated with BSJPD (one decoction a day) after TACE treatment. In both of the groups, the patients underwent ≥ 12 weeks of treatment.

The inclusion criteria included the following: primary carcinoma of the liver; over 18 years of age; received a treatment of transcatheter arterial chemoembolization (TACE); and one month without radiotherapy or chemotherapy. The following exclusion criteria were used: metastatic hepatic carcinoma; serious complications (hepatic failure, renal failure, etc.); or incomplete medical records.

The patient survival time was calculated by multivariate Cox regression analyses. Sample size: n_t_ = n_c_ = 37. Sample size was calculated by SPSS 18.0 (*α*: 0.05, *β*: 0.2, M_t_: 12, M_c_: 6, T_0_: 6, T: 30).

Additional information regarding this study includes the following: entitled Study of TCM Syndrome of Hepatocellular Carcinoma and Colorectal Cancer Based on System Science; ClinicalTrials.gov Identifier is NCT03189992; date of registration was June 16, 2017; retrospectively registered. All experimental protocols were approved by the IRB of the Shuguang Hospital affiliated with the Shanghai University of Traditional Chinese Medicine (2014-345-41-01) in accordance with the Guidelines for Ethical Review of Drug Clinical Trials, the Declaration of Helsinki, the International Ethical Guidelines for Biomedical Research Involving Human Subjects (CIOMS: 2002).

### 2.5. Collection of the Herbal Formulation Compounds and Targets

The chemical compounds and the targets of the compounds were identified using the Traditional Chinese Medicine (TCM) database at Taiwan (http://tcm.cmu.edu.tw/), the TCM Systems Pharmacology (TCMSP) database (http://lsp.nwu.edu.cn/tcmsp.php) and PDTCM (http://cadd.gdhtcm.com:2180/PDTCM/index.php) [15]. The compounds were screened for oral bioavailability (OB) > 30 % and drug-likeness (DL) > 0.18). The liver cancer-related targets were collected from GeneCards (https://www.genecards.org/) and the OMIM database (www.omim.org/); the screening criteria were liver cancer, highlights, and scores > 25.

### 2.6. Network Pharmacology Analysis and Enrichment Analysis

The compound-target networks of BSJPD were analyzed by the Cytoscape software (version 3.2.0). The related parameters [16] were calculated to explore the significant nodes. The screening criteria were the degree, betweenness-centrality, and closeness-centrality values that were greater than the average values when constructing a major network. All pathways associated with BSJPD and the liver were obtained by KEGG.

### 2.7. MTS Assay

As previously described [17], HepG2 cells were cultured in a 96-well plate (5.0*∗*10^3^ cells/well) for 12 h, followed by incubation with quercetin, kaempferol, hederagenin, *β*-sitosterol, stigmasterol, formononetin, isorhamnetin, alisol B, or licochalcone A (6.25-200 *μ*mol/l) for 24 h or with licochalcone A, alisol B, hederagenin (5, 10, 20 *μ*mol/l), or BSJPD-medicated serum (5 %, 10 %, and 20 %) at 37°C, to observe their effects on cell viability. CellTiter AQ solution (20 *μ*l; containing MTS) was added to each well. After 4 h, the absorbance at 490 nm was measured, and the cell viability and IC50 values were calculated. All assays were repeated at least 3 times.

### 2.8. Cell Apoptosis Detection

HepG2 cells were cultured in a 6-well plate (3*∗*10^5^ cells/well) for 12 h followed by incubation with alisol B (5 *μ*mol/l), licochalcone A (10 *μ*mol/l), hederagenin (10 *μ*mol/l), TNF-*α* (20 ng/mL), or BSJPD-medicated serum (20 %) for 24 h. A single cell suspension (total 1*∗*10^6^ cells) was prepared from the fresh liver cancer tissues of mice. One gram of fresh liver cancer tissue was smashed and washed with PBS, filtered through a 75 *μ*M filter membrane, and then fixed in 70 % ethanol at 4°C for 12 h. Then, a Muse™ Annexin V Dead Cell Kit was used for apoptosis detection. The assays were repeated 3 times. The total apoptotic rate was calculated by the following formula: The total apoptotic rate = apoptotic cells/total cells ×100%.

### 2.9. Western Blot Assay

HepG2 cells were cultured in a 6-well plate with a density of 1.0*∗*10^6^ cells/well for 12 h followed by incubation with alisol B (5 *μ*mol/l), licochalcone A (10 *μ*mol/l), hederagenin (10 *μ*mol/l), or BSJPD-medicated serum (20 %) for 48 h. Then, the cells were collected and assayed as described in previous literature [17], and assays were repeated at least 3 times. The full-length gels and blots are included in the Supplementary Figures.

### 2.10. Statistical Analysis

The one-way analysis of variance (ANOVA), rank-sum test, Kaplan-Meier curves, and multivariate Cox regression analysis were performed (SPSS 18.0 software).* p* < 0.05 was considered significant.

## 3. Results

### 3.1. Patient Characteristics and Survival Analysis

**Figure 1:**
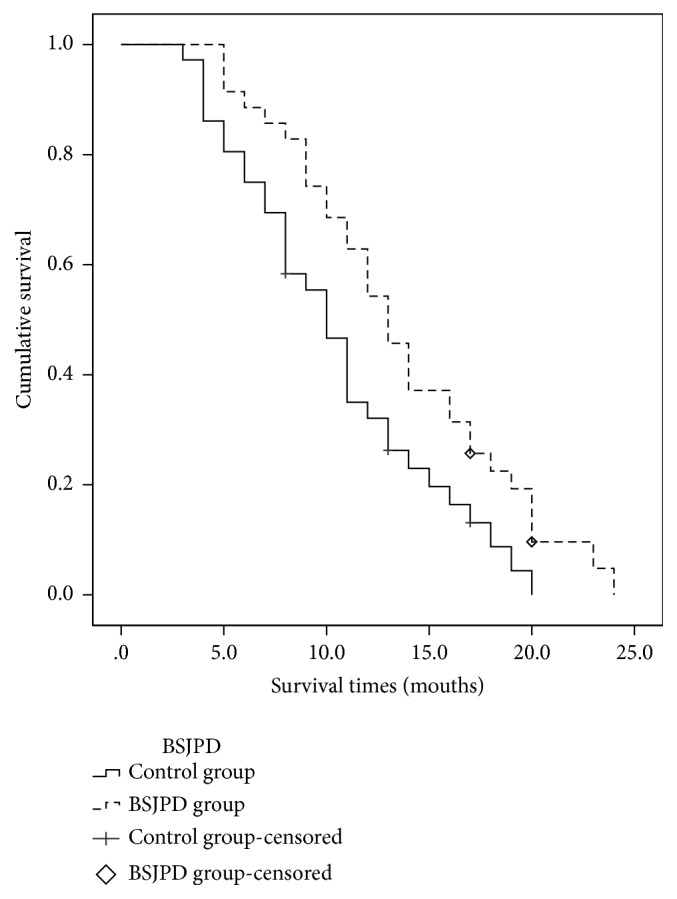
Survival analysis between the control and BSJPD group.* Notes. *The median overall survival in the BSJPD group was longer than that in the control group (11 versus 8 months; p =0.016,* p* <0.05). BSJPD, Bushen-Jianpi decoction.

Overall, 71 patients (5 were lost to follow-up) had LC in this study. A total of 49.3 % patients were using BSJPD. The univariate analysis revealed that clinical stage composition IV was a risk factor associated with the median overall survival (*p* < 0.05). The Cox regression analysis revealed that BSJPD was an independent protective factor for survival (Tables 1 and 2). The median survival time of the BSJPD group was longer than that of the control group (Figure 1). Most patients were males because males are more often affected by LC [18]. In this work, gender was not a protective factor or risk factor for survival. Thus, gender had no markedly effect in this study. The baseline data are shown in Supplementary Table 1. These results showed that BSJPD can improve patient survival.

### 3.2. Compounds and Targets of BSJPD

**Figure 2:**
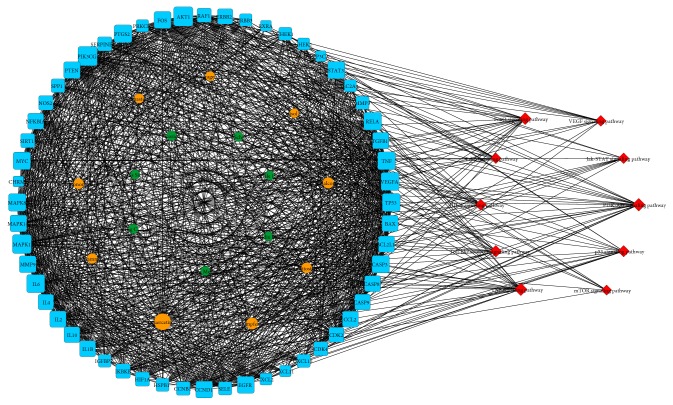
Herb-compound-target-signaling pathway network of BSJPD.* Notes. *The green octagon nodes represent herbs, the orange ellipse nodes represent compounds, the blue square nodes represent targets, and the red diamond nodes represent signaling pathways. The sizes of the nodes are illustrated from large to small in descending order of degree values. BSJPD, Bushen-Jianpi decoction.

The BSJPD network was constructed for screening the important compounds and targets of BSJPD. After the evaluation of the network parameters, the major compound-target signaling pathway networks of the herbs in BSJPD were constructed (Figure 2). This network includes 82 nodes and contains 7 herbs, 9 compounds, 56 targets, and 10 pathways. The sizes of the nodes represent the degree values, and larger sized nodes indicate more importance. These compounds and targets are listed in Supplementary Table 2.

### 3.3. Putative Pharmacological Mechanisms of BSJPD

**Figure 3:**
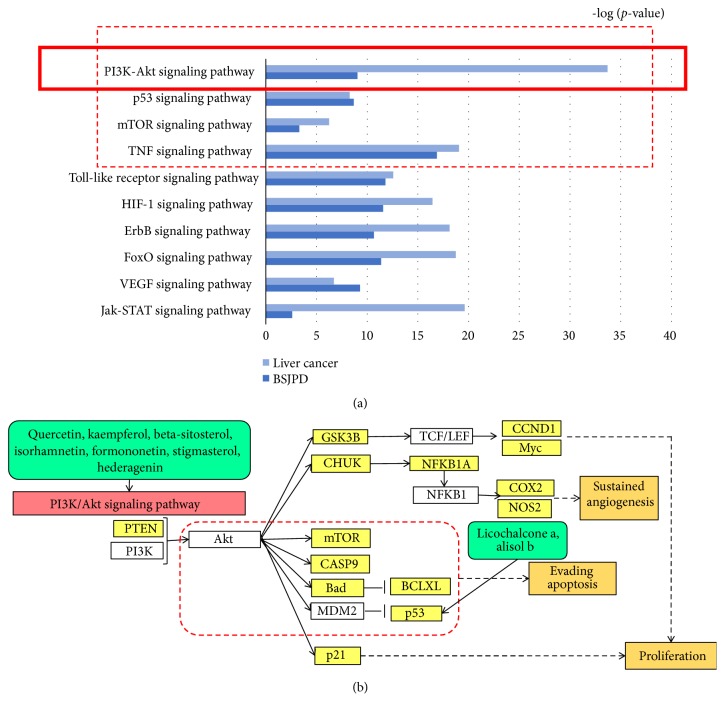
Putative signaling pathways of BSJPD. (a) Top 10 shared signaling pathways of LC and BSJPD. (b) Putative major signaling pathways of BSJPD.* Notes. *The rounded rectangles represent compounds, the gray squares represent validated targets, the white squares represent predicted targets, the trapezoids represent signaling pathways, and the ellipses represent biological process. BSJPD, Bushen-Jianpi decoction. LC, liver cancer.

In total, 3148 targets of liver cancer were identified. All BSJPD and LC pathways were obtained. The LC-related signaling pathways shared by LC and BSJPD are shown in Figure 3. After the comprehensive evaluation of the topological parameters and shared signaling pathways, 9 compounds were selected. Putative major signaling pathways of BSJPD were constructed. The top signaling pathways were considered in the experimental verification.

### 3.4. BSJPD-Medicated Serum and Its Major Compounds Inhibited the Viability of HepG2 Cells

**Figure 4:**
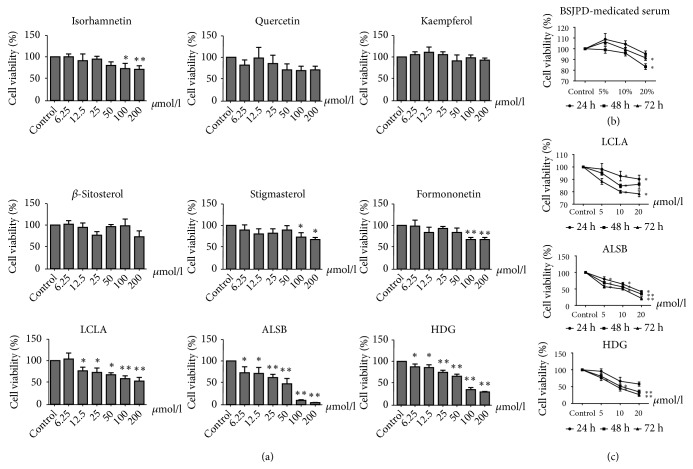
BSJPD-medicated serum and its major compounds inhibited the viability of HepG2 cells. (a) Effects of BSJPD-medicated serum on HepG2 cell viability at 24, 48, and 72 h. (b) Effects of quercetin, kaempferol, *β*sitosterol, stigmasterol, formononetin, isorhamnetin, licochalcone A, alisol B, and hederagenin on HepG2 cell viability at 24 h. (c) Effects of licochalcone A, alisol B, and hederagenin on HepG2 cell viability at 24, 48, and 72 h.* Notes. *BSJPD, Bushen-Jianpi decoction; LCLA, licochalcone A; ALSB, alisol B; HDG, hederagenin. *∗ p* < 0.05; *∗∗ p* < 0.01, compared with the control group.

In Figure 4, 20 % BSJPD-medicated serum significantly inhibited the viability of HepG2 cells at 48 h and 72 h (*p* < 0.05). No obvious effects on HepG2 cells of the major compounds other than low concentrations of licochalcone A, alisol B, and hederagenin were observed (*p* < 0.05; Figure 4(a)). HepG2 cell viability was markedly decreased by licochalcone A, alisol B, and hederagenin at 48 h and 72 h (*p* < 0.05; Figure 4(c)). The IC50 values of the 9 major compounds are shown in Table 3. Licochalcone A, alisol B, and hederagenin showed better effects on inhibiting viability. These results showed that licochalcone A, alisol B, and hederagenin have antitumor effects. The viability was observed at 24 h, 48 h, and 72 h to find a more effective compounds treatment time. Thus, 48 h as a BSJPD-medicated serum and compounds treatment time was chosen to make western blot assay.

### 3.5. Effect of BSJPD on Total Apoptotic Rate of LC Tissues in Mice with Transplanted Primary Hepatocellular Carcinoma

**Figure 5:**
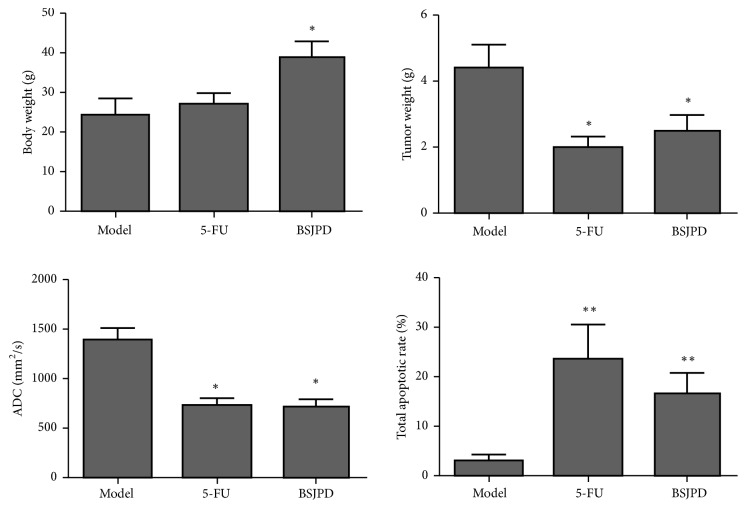
Effect of BSJPD on total apoptotic rate of LC tissues in mice with transplanted primary hepatocellular carcinoma.* Notes.* n = 12. ADC, apparent diffusion coefficient. 5-FU, 5-Fluorouracil. BSJPD, Bushen-Jianpi decoction. LC, liver cancer. *∗p* < 0.05; *∗∗p* < 0.01, compared with model group.

Compared to the model mice, BSJPD treatment increased the apoptotic rate in LC tissues and reduced the tumor weight and the apparent diffusion coefficient. As a positive control, 5FU treatment increased the total apoptotic rate of the LC tissues and reduced the tumor weight and the apparent diffusion coefficient, as shown in Figure 5. These results indicated that BSJPD can induce apoptosis in LC tissues.

### 3.6. BSJPD-Medicated Serum, Licochalcone A, Alisol B, and Hederagenin Induced Apoptosis in HepG2 Cells

**Figure 6:**
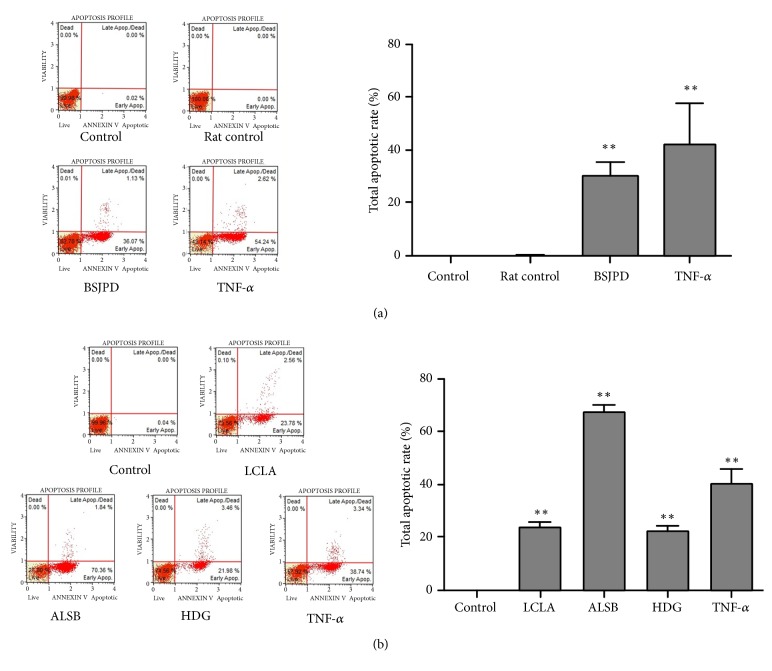
Effects of BSJPD-medicated serum, licochalcone A, alisol B, and hederagenin on HepG2 cell apoptosis.* Notes.* BSJPD, Bushen-Jianpi decoction; LCLA, licochalcone A; ALSB, alisol B; HDG, hederagenin. *∗ p *< 0.05; *∗∗ p* < 0.01, compared with the control group.

The results of the Muse™ Annexin V Dead Cell Kit analysis are displayed in the histograms shown in Figure 6. The total apoptotic rates were increased in response to BSJPD-medicated serum, licochalcone A, alisol B, and hederagenin (*p* < 0.01). BSJPD-medicated serum, licochalcone A, alisol B, and hederagenin showed marked effects on apoptosis induction. These results indicated that BSJPD and licochalcone A, alisol B, and hederagenin can induce apoptosis in HepG2 cells.

### 3.7. Effects of BSJPD-Medicated Serum, Licochalcone A, Alisol B, and Hederagenin on the Apoptosis-Related Pathways in HepG2 Cells

**Figure 7:**
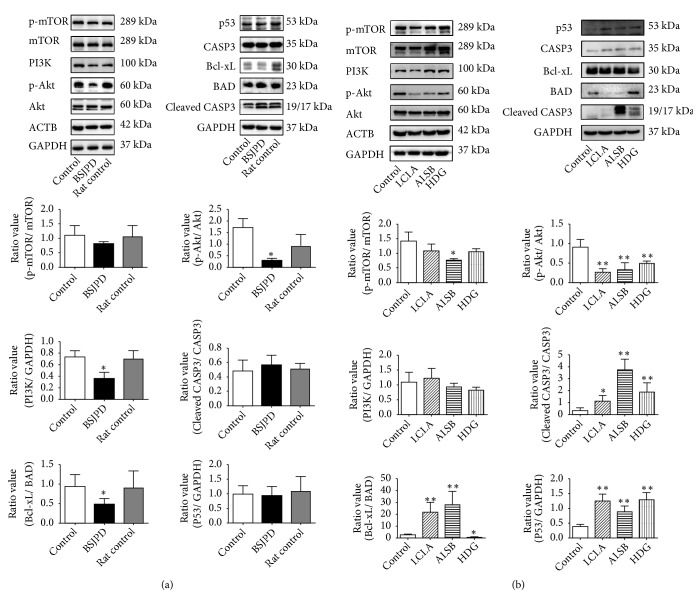
Effects of BSJPD-medicated serum, licochalcone A, alisol B, and hederagenin on the expression of PI3K-Akt-mTOR pathway components and apoptosis-related pathway molecules in HepG2 cells. (a) Western blot analysis of the effects of BSJPD-medicated serum on the expression of PI3K, p-Akt, Akt, p-mTOR, mTOR, Bcl-xL, BAD, p53, and CASP3. (b) Western blot analysis of the effects of licochalcone A, alisol B, and hederagenin on the expression of PI3K, p-Akt, Akt, p-mTOR, mTOR, Bcl-xL, BAD, p53, and CASP3.* Notes.* BSJPD, Bushen-Jianpi decoction; LCLA, licochalcone A; ALSB, alisol B; HDG, hederagenin; control, cultured with FBS; rat control, cultured with rat serum. *∗ p* < 0.05 and *∗∗ p* < 0.01 compared with the control group. These groupings of blots are cropped from the same gel or different gels. All full-length blots are included in Supplementary Figures 3 and 4.

An evaluation of the apoptosis-related pathways, revealing the mechanism of BSJPD, is shown in Figure 7. The BSJPD-medicated serum dramatically decreased the expression of PI3K, Bcl-xL/BAD, and p-Akt (*p* < 0.05); there was a trend of p-mTOR expression downregulation (Figure 7(a)). The following results are shown in Figure 7(b): licochalcone A and alisol B increased the level of Bcl-xL/BAD; alisol B and hederagenin increased the expression of cleaved-CASP3/CASP3; hederagenin reduced the level of Bcl-xL/BAD; alisol B decreased the expression of p-mTOR; and all treated groups resulted in a decrease in p-Akt expression and an increase in p53 expression. BSJPD-medicated serum, licochalcone A, alisol B, and hederagenin showed marked effects on the PI3K-Akt and apoptosis-related pathways. These results indicated that BSJPD and licochalcone A, alisol B, and hederagenin can regulate the apoptosis-related pathways in HepG2 cells.

## 4. Discussion

In the clinic, LC patients often have poor prognoses. Most studies have focused on systemic chemotherapeutic drugs [19–23]. However, the strategy of therapy has been ineffective in promoting survival and improving prognosis. Advanced stage LC is still an incurable disease. According to statistics, the median survival time is approximately 6 months [6]. Currently, the objective of therapy for this cancer is to prolong the overall survival. However, the benefits of TCM treatments seem to be promising in LC [2], breast cancer [7], etc. Thus, clarifying the anticancer mechanism of action of TCM treatment is important. In our study, correlation analysis revealed that BSJPD was an independent protective factor for survival. BSJPD prolonged survival in patients with LC. These results indicate that the use of BSJPD for LC treatment was effective. We further investigated the mechanism of action of BSJPD with network pharmacology analysis and experimental verification. Network pharmacology can identify potentially active compounds, targets, and pharmacological mechanisms of complex compounds in Chinese herbal formulas [7, 24, 25]. In this work, we identified 143 compounds in the 9 herbs of BSJPD and 249 related targets. Furthermore, 9 compounds, 56 targets, and 10 pathways were selected by structuring a compound-target network of BSJPD to select the major compounds and targets using topological parameters. Among these, quercetin can inhibit the proliferation of malignancies [26], and kaempferol has anticancer activity [27]. These top targets and pathways are also related to LC processes [28]. Therefore, BSJPD could affect LC via these compounds, targets and pathways. In view of the results of the enrichment analysis, we found that the LC signaling pathways impacted by treatment with BSJPD mostly involved tumor apoptosis and growth, such as the PI3K-Akt-mTOR, p53, TNF, and VEGF pathways [28, 29]. Thus, after evaluating the top shared signaling pathways and major networks, we constructed a putative major signaling pathway of BSJPD.

From this network pharmacology study, we hypothesized that BSJPD could promote apoptosis in LC by regulating apoptosis-related pathways, leading to an increase in survival. The effects of BSJPD on these pathways may be derived from the microsynergism of the 9 major compounds. These compounds can affect different targets in these pathways at the same time. However, distinguishing the effects of activation and inhibition is difficult when using network pharmacology. Moreover, this approach is susceptible to influence from different prediction tools. Therefore, it is essential to validate the prediction.

As a special cell population, tumors are closely related to overproliferation and the inhibition of apoptosis [30]. Thus, the cell viability assay is commonly used in evaluating the efficacy of anticancer drugs. In our work, a reduction in cell viability was observed in the group treated with 20 % BSJPD-medicated serum. With the 9 major compounds, no significant changes in cell viability were found at low concentrations in most groups, but a reduction in HepG2 cell viability was observed with the licochalcone A, alisol B, and hederagenin treatments at multiple time points. These results showed that BSJPD and its major compounds can inhibit HepG2 cell survival. Licochalcone A, alisol B, and hederagenin can inhibit tumor cell viability. In addition, previous studies have reported that the action of some compounds in BSJPD such as kaempferol may influence the cell cycle [31]. Formononetin alleviates the patient's pain and enhances immunity [32] and transresveratrol and quercetin improve nutrition [33]. Furthermore, according to the network pharmacology prediction, BSJPD may be associated with apoptosis processes.

Apoptosis, an important biological process in tumor growth, is the programmed cell death process in multicellular organisms [34, 35]. Apoptosis is generally suppressed in tumor cells. Inducing apoptosis can inhibit the tumor process [36]. Thus, apoptosis plays a vital role in LC treatment. Our results from the apoptosis assay show that an increase in the total apoptosis rate occurred in the fresh LC tissues of the BSJPD-treated mouse group with transplanted primary hepatocellular carcinoma. The increase also occurred in the BSJPD-medicated serum-, licochalcone A-, alisol B-, and hederagenin-treated groups. These data indicated that BSJPD and its major compounds can induce apoptosis, which is in line with our prediction. Through the downstream events of intracellular signaling cascades, apoptosis can be induced by the activation and inhibition of several signaling pathways. Additionally, in our prediction, BSJPD mainly regulates some apoptosis-related pathways to promote apoptosis. The overexpression of the PI3K-Akt-mTOR pathway [37, 38] can promote the migration and proliferation as well as inhibiting the apoptosis of hepatoma cells, which is related to highly reduced survival [29, 39]. Some cytokines, such as EGF and insulin, can also activate the PI3K-Akt-mTOR pathway [38]. PI3K activates Akt by PIP3 [40]. Phospho-Akt can affect its substrates, including mTOR, BAD, and NF-kB, leading to the direct or indirect regulation of proliferation and the inhibition of apoptosis [30]. Phospho-mTOR, which is activated by Akt, can promote proliferation and indirectly protect tumor growth [37]. Thus, downregulating this overexpression may rebuild the balance of proliferation and apoptosis in hepatoma cells, leading to increased survival [41, 42]. In the present study, we demonstrate the alterations in PI3K, p-Akt, Akt, p-mTOR, and mTOR after BSJPD treatment and treatment with its major compounds. The decreases in PI3K and p-Akt caused by BSJPD-medicated serum demonstrate that the BSJPD-treated HepG2 cells experience a downregulation in PI3K-Akt-mTOR pathway signaling. The reductions in p-Akt and p-mTOR expression in the major compound groups showed that licochalcone A, alisol B, and hederagenin mainly inhibit phospho-Akt and phospho-mTOR. These results suggest that the differences in the results of the BSJPD treatment and the treatments with its major compounds are due to the effects of the compounds on different targets, such as only ALSB decreased p-mTOR/mTOR ratio, HDG decreased Bcl-xL/BAD ratio, ALSB, and LCLA increased Bcl-xL/BAD ratio. Although the major compounds affect different targets, their comprehensive effects on the PI3K-Akt-mTOR pathway are consistent with those of BSJPD treatment as well as the results of the network pharmacology prediction, indicating that BSJPD and its major compounds induce apoptosis and may be involved in regulating the PI3K-Akt-mTOR pathway.

Apoptosis is inhibited in the LC process [6, 38]. CASP3 is one of the caspase proteins. As one of the classic markers of apoptosis, CASP3 is activated by death ligands [43, 44]. Because this protein is activated by cleaving, the quantification of cleaved-CASP3 can accurately assess apoptosis. The tumor suppressor p53 can suppress cancer progression by activating apoptosis, activating DNA repair proteins, and maintaining genomic stability [45, 46]. Increasing and promoting the endogenous recovery of p53 expression has been seen as a benefit for cancer therapy. Therefore, assaying p53 expression is vital to assessing the effects of anticancer drugs. Bcl-xL can inhibit the activation of caspases by suppressing the release of cytochrome c, leading to an antiapoptotic effect [47]. In contrast, BAD protects the process of apoptosis by forming a heterodimer with Bcl-xL [48]. Changes in Bcl-xL and BAD expression are usually considered indicators for beneficial HCC treatment [49, 50]. Thus, the Bcl-xL/BAD ratio and their individual levels are suitable parameters to evaluate the effect of BSJPD. In this study, the complex results of the treatment with BSJPD and its individual major compounds are shown. The stimulatory and inhibitory effects of the major compounds exist simultaneously, explaining the effects of the compounds on different targets. Even so, the increase in p53 and cleaved-CASP3 levels caused by the 3 major compounds is very clear; thus, the major compounds represent the comprehensive effect of BSJPD and determine the level of apoptosis. The results suggest that BSJPD and its major compounds can induce apoptosis by regulating apoptosis-related factors, including p53, CASP3, and Bcl-xL/ BAD, as well as the PI3K-Akt-mTOR pathway.

## 5. Conclusions

Overall, this work proves that BSJPD can prolong the survival of patients with LC and promote hepatoma cell apoptosis, which is connected to its modulation of the PI3K-Akt-mTOR pathway and the p53, CASP3, and Bcl-xL/BAD proteins. These effects may be partly derived from licochalcone A, alisol B, and hederagenin, which are 3 major compounds in the network pharmacology prediction. However, the synergistic effects and the metabolites of these compounds were not investigated in this work. In a future study, the microsynergistic effects of the multiple compounds in BSJPD and the relationship between the effects of these compounds and their metabolic mechanisms will be investigated.

## Figures and Tables

**Table 1:** Univariate analysis of 71 patients with hepatic carcinoma.

Characteristics	Univariate analysis	
	N (%)	P-value
Gender		0.931
male	63 (88.7)	
female	8 (11.3)	
Age		0.479
≥50	47 (66.2)	
< 50	24 (33.8)	
Clinical stage composition		0.023
I-III	31 (43.7)	
IV	40 (56.3)	
Serum IL10 levels		0.916
> 23.35 pg/mL	43 (60.6)	
< 23.35 pg/mL	18 (25.4)	
Serum IL12-P40 levels		0.345
> 200 ng/L	63 (88.7)	
< 200 ng/L	8 (11.3)	
Serum IFN-*γ* levels		0.285
> 15 *μ*g/L	53 (74.6)	
< 15 *μ*g/L	18 (25.4)	
BSJPD		0.006
yes	35 (49.3)	
no	36 (50.7)	

*Notes. *BSJPD, Bushen-Jianpi decoction; IL, interleukin; IFN-*γ*, interferon-*γ*; TACE, transcatheter arterial chemoembolization.

**Table 2:** Multivariate analysis of 71 patients with hepatic carcinoma.

Characteristics	Multivariate analysis					
	N (%)	B	*β*	Exp (*β*)	95% CI for Exp(B)	P-value
Gender		-	-	-	-	0.53
male	63 (88.7)					
female	8 (11.3)					
Age		-	-	-	-	0.768
≥50	47 (66.2)					
< 50	24 (33.8)					
Clinical stage composition		2.756	0.696	2.006	1.165-3.455	0.012
I-III	31 (43.7)					
IV	40 (56.3)					
Serum IL10 levels		-	-	-	-	0.843
> 23.35 pg/mL	43 (60.6)					
< 23.35 pg/mL	18 (25.4)					
Serum IL12-P40 levels		-	-	-	-	0.335
> 200 ng/L	63 (88.7)					
< 200 ng/L	8 (11.3)					
Serum IFN-*γ* levels		-	-	-	-	0.214
> 15 *μ*g/L	53 (74.6)					
< 15 *μ*g/L	18 (25.4)					
BSJPD		3.258	- 0.567	0.567	0.337-0.954	0.032
yes	35 (49.3)					
no	36 (50.7)					

*Notes. *BSJPD, Bushen-Jianpi decoction; IL, interleukin; IFN-*γ*, interferon-*γ*; TACE, transcatheter arterial chemoembolization.

**Table 3:** IC 50 values of 9 major compounds with HepG2 cell lines.

Compound	IC 50 (*μ*mol/l) HepG2
Quercetin	> 200
Kaempferol	> 200
Hederagenin	72.37
*β*-sitosterol	> 200
Stigmasterol	> 200
Formononetin	> 200
Isorhamnetin	> 200
Alisol B	32.57
Licochalcone a	127.90

## Data Availability

All data generated or analyzed during this study are included in this published article and supplementary materials.

## Abbreviations

BSJPD: Bushen-Jianpi decoction
LC: Liver cancer
5-FU: 5-Fluorouracil
LCLA: Licochalcone A
ALSB: Alisol B
HDG: Hederagenin
TCM: Traditional Chinese medicine
LWDHD: Liu-Wei-Di-Huang decoction
SJZD: Si-Jun-Zi decoction
TACE: Transcatheter arterial chemoembolization
PI3K: Phosphoinositide 3-kinase
Akt: Protein kinase B
mTOR: Mammalian target of rapamycin
p53: Tumor protein p53
CASP3: Caspase-3
Bcl-xL: B-cell lymphoma-extra large
BAD: Bcl-2-associated death promoter
GAPDH: Glyceraldehyde 3-phosphate dehydrogenase
ACTB: Beta-actin.

## Supplementary Materials

Supplementary 1 Supplementary Figure 1: chemical structures. Supplementary Figure 2: determination of morroniside, loganin, and paeonol in BSJPD extracts and BSJPD-medicated serum sample by HPLC-MS MRM. Supplementary Figures 3 and 4: all full-length western blots.

Supplementary 2 Supplementary Table 1: patients' baseline characteristics and treatments between the control group and the BSJPD group.

Supplementary 3 Supplementary Table 2: all ingredients and targets of BSJPD.
