# Supplementary material for: Network Pharmacology-Based Study on the Mechanism of Bushen-Jianpi Decoction in Liver Cancer Treatment
Source: Evid Based Complement Alternat Med. 2019 Mar 19;2019:3242989. doi: 10.1155/2019/3242989 (PMC6444272; doi:10.1155/2019/3242989)

Network pharmacology-based study on the mechanism of Bushen-Jianpi decoction in liver cancer treatment

Bushen-Jianpi decoction in liver cancer treatment

Rong Wu[1]*, Xiao-Yan Li[1]*, Wen-Hai Wang[2]*, Fei-Fei Cai[1], Xiao-Le Chen[1], Meng-Die Yang[1], Qiu-Sha Pan[1], Qi-Long Chen1, Rong-Yao Zhou[3], Shi-Bing Su [1]**

[1] Research Center for Traditional Chinese Medicine Complexity System, Shanghai University of Traditional Chinese Medicine, Shanghai, 201203, China
[2] Shanghai Baoshan Hospital of Integrated Traditional Chinese Medicine and Western Medicine, Shanghai University of Traditional Chinese Medicine, Shanghai 201999, China.
[3] Department of Medical Oncology, Shuguang Hospital, Shanghai University of Traditional Chinese Medicine, Shanghai 201203, China.
* co-first author.

** Correspondence: Shi-Bing Su, shibingsu07@163.com.

1. Supplementary Figures

1.1 Supplementary Figure 1

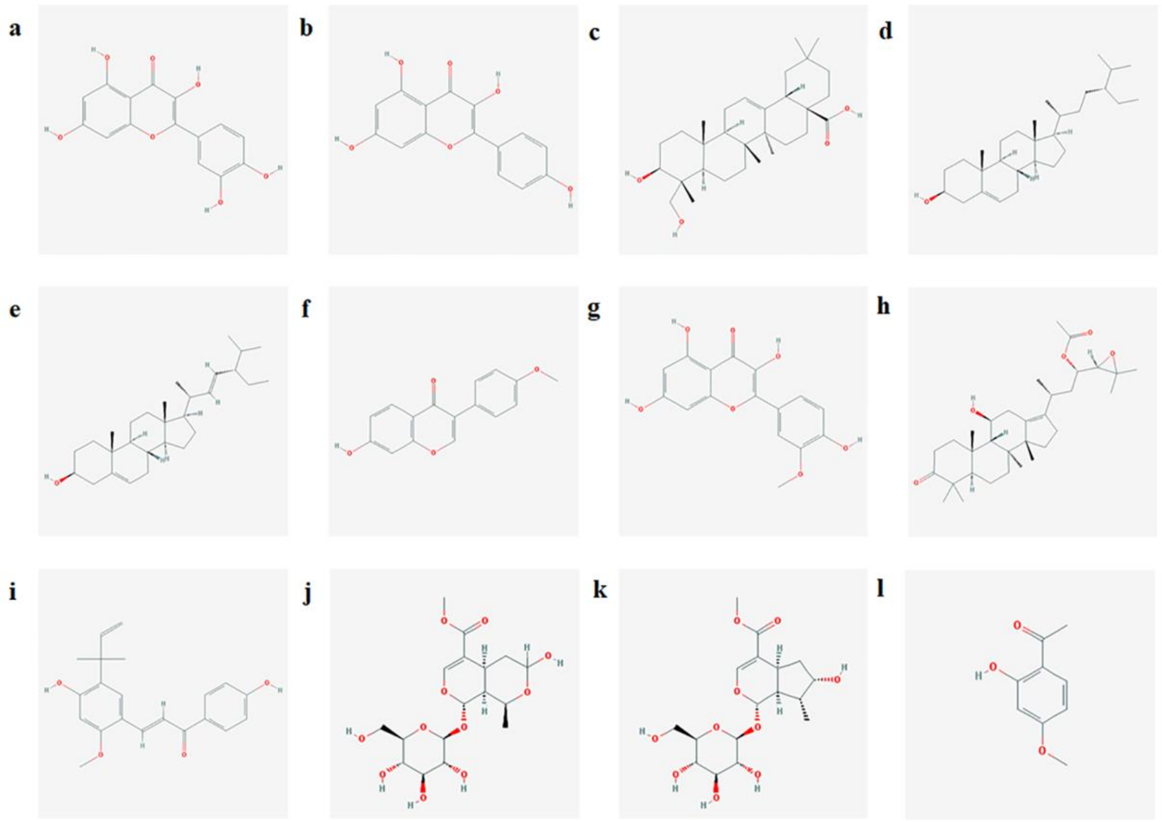

Supplementary Figure 1: Chemical structures of quercetin, kaempferol, hederagenin, β-sitosterol, stigmasterol, formononetin, isorhamnetin, alisol B and licochalcone A. a, quercetin; b, kaempferol; c, hederagenin; d, β-sitosterol; e, stigmasterol; f, formononetin; g, isorhamnetin; h, alisol B; i, licochalcone A; j, Morroniside; k, Loganin; l, paeonol.

1.2 Supplementary Figure 2

HPLC-MS conditions: Accela High performance liquid chromatography and LTQ Orbitrap XL were purchased from Thermo Fisher Scientific Company (USA); methanol (HPLC Grade) and formic acid (HPLC Grade) were purchased from Thermo Fisher Scientific Company (USA); reference standards were obtained from the Shanghai Institute of Materia Medica, Chinese Academy of Sciences.

BSJPD extracts and BSJPD-medicated serum sample were performed on high performance liquid chromatography (HPLC) Accela600 pump, LTQ Orbitrap XL (Thermo Fisher Scientific Company, USA) using a SBAq column (4.6 × 250mm, 5 micron, Agilent Technologies, USA), Capillary Voltage 2500V–3000V, Tubeleu110V, Scan range100–1500, Sheath Gas 30 psi, and Aux Gas Flow 10 psi.

Method: The mobile phases comprised eluent A (0.1%formic acid) and eluent B (methanol). The gradient flow was as follows: 0~5minutes, 30% B; 5~40minutes, 30–90% B; 40~45

minutes, 90% to 100% B; 45~50minutes, 100% B. The analysis was performed at a flow rate of 1.0mL/min. The injection volumewas10μl.

Sample preparation: BSJPD extracts were diluted into a concentration of 1mg/mL by 50% methanol-water solution, then the mix solution was ultrasonic extracted for 30 minutes at room temperature, filtered at 0.22 μm filter, stored at 4 ℃. About 200 μl of the serum and 400 μl of acetonitrile were mixed and vortexed for 30 s. After centrifugation at 13,000 rpm for 10 min at 4 ℃. The concentrations of three compounds were 1mg/ml.

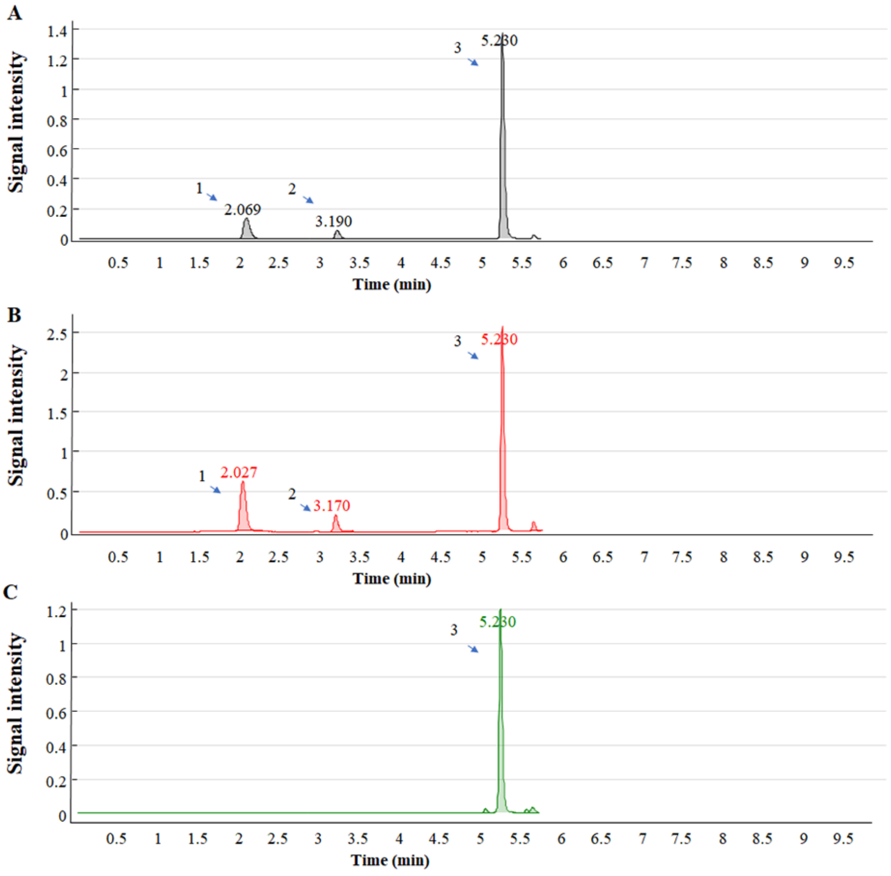

PEAK (a)	Area count
1	74770.32
2	23261.01
3	432798

PEAK (b)	Area count
1	31127.58
2	7708.85
3	74609.31

PEAK (c)	Area count
1	/
2	/
3	33720.59

Supplementary Figure 2: Determination of morroniside, loganin and paeonol in BSJPD extracts and BSJPD-medicated serum sample by HPLC-MS MRM. a, morroniside, loganin and paeonol control; b, BSJPD extracts; c, BSJPD-medicated serum sample. *Notes.* 1, 2 and 3 represents morroniside, loganin and paeonol.

1.3 Supplementary Figure 3

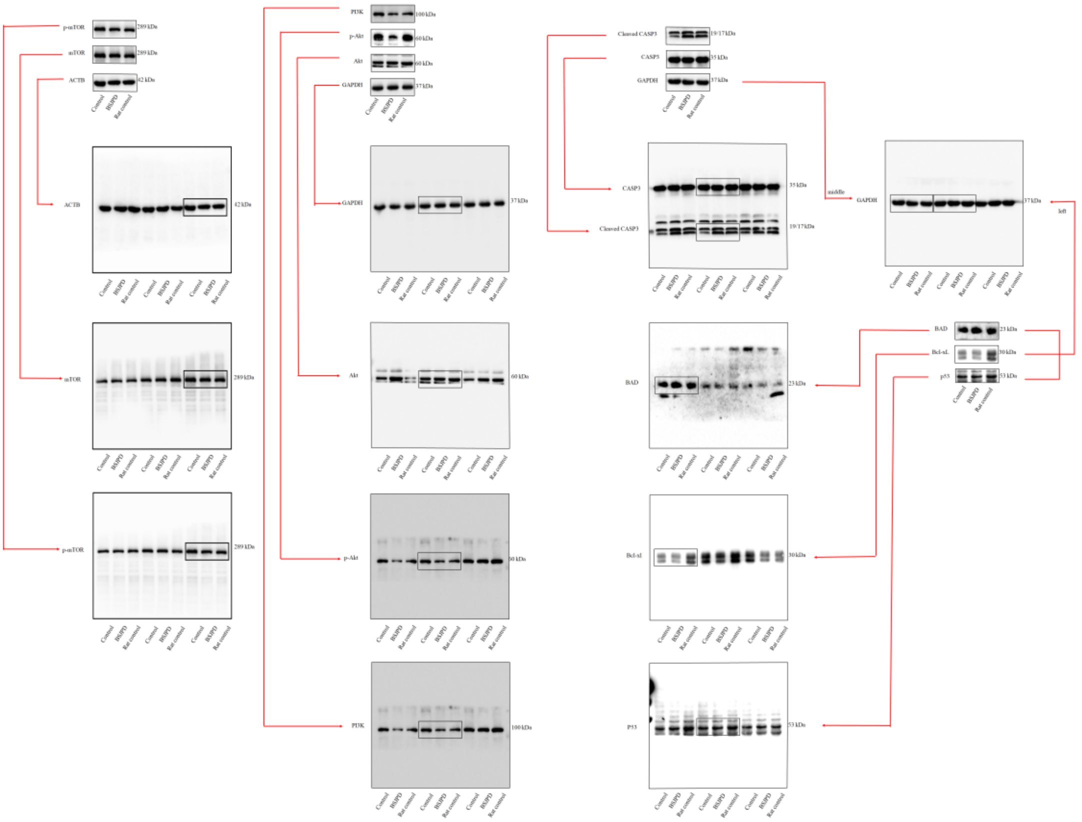

1.3 Supplementary Figure 4.

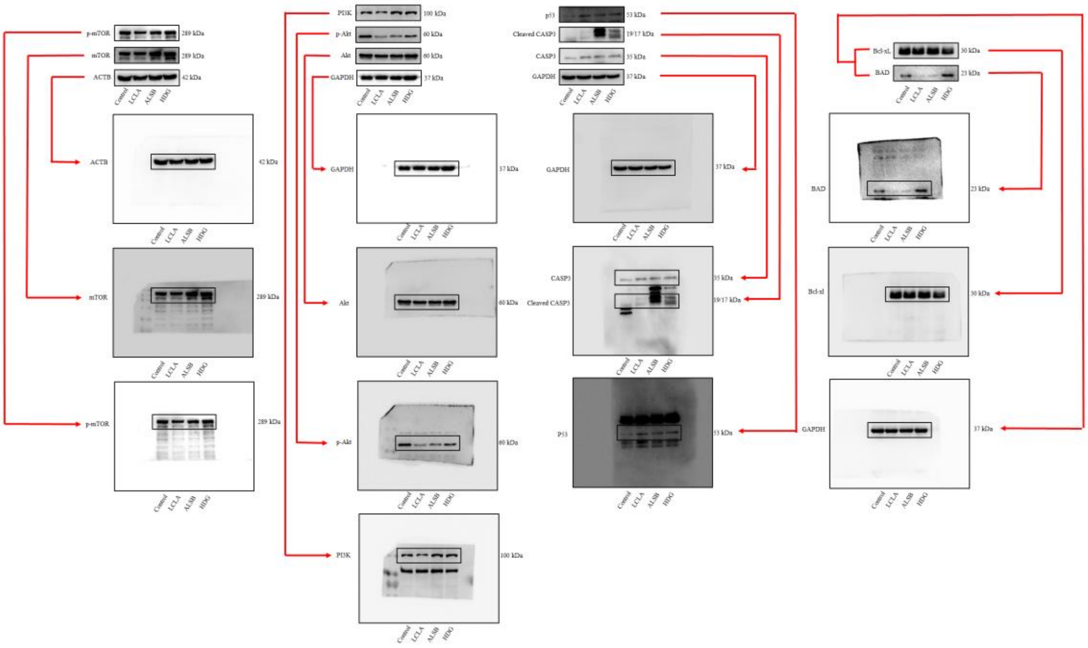

Supplement: Supplementary 1 — Supplementary Figure 1: chemical structures. Supplementary Figure 2: determination of morroniside, loganin, and paeonol in BSJPD extracts and BSJPD-medicated serum sample by HPLC-MS MRM. Supplementary Figures 3 and 4: all full-length western blots. [file 3242989.f1.pdf]
