# Supplementary material for: Network Pharmacology-Based Study on the Mechanism of Bushen-Jianpi Decoction in Liver Cancer Treatment
Source: Evid Based Complement Alternat Med. 2019 Mar 19;2019:3242989. doi: 10.1155/2019/3242989 (PMC6444272; doi:10.1155/2019/3242989)
Supplement: Supplementary 2 — Supplementary Table 1: patients' baseline characteristics and treatments between the control group and the BSJPD group. [file 3242989.f2.pdf]

Network pharmacology-based study on the mechanism of Bushen-Jianpi decoction in liver cancer treatment

Bushen-Jianpi decoction in liver cancer treatment

Rong Wu[1]*, Xiao-Yan Li[1]*, Wen-Hai Wang[2]*, Fei-Fei Cai[1], Xiao-Le Chen[1], Meng-Die Yang[1], Qiu-Sha Pan[1], Qi-Long Chen1, Rong-Yao Zhou[3], Shi-Bing Su [1]**

[1] Research Center for Traditional Chinese Medicine Complexity System, Shanghai University of Traditional Chinese Medicine, Shanghai, 201203, China
[2] Shanghai Baoshan Hospital of Integrated Traditional Chinese Medicine and Western Medicine, Shanghai University of Traditional Chinese Medicine, Shanghai 201999, China.
[3] Department of Medical Oncology, Shuguang Hospital, Shanghai University of Traditional Chinese Medicine, Shanghai 201203, China.
* co-first author.

** Correspondence: Shi-Bing Su, shibingsu07@163.com.

1. Supplementary Tables

1.1 Supplementary Table 1

Supplementary Table 1: Patients' baseline characteristics and treatments between the control group and the BSJPD group.

t variable	Control group (n = 36)	BSJPD group (n = 35)	P-value
Age (≥ 50/ < 50)	24/12	23/12	0.932
Gender (male/ female)	31/5	32/3	0.479
Clinical stage composition (I-III/ IV)	15/21	16/19	0.746
Serum IL10 levels (> 23.35 pg/mL/ < 23.35 pg/mL)	21/15	22/13	0.628
Serum IL12-P40 levels (> 200 ng/L/ < 200 ng/L)	31/5	32/3	0.479
Serum IFN-γ levels (> 15 μg/L/ < 15 μg/L)	28/8	25/10	0.709
TACE (yes/ no)	36/0	35/0	-

Notes. BSJPD, Bushen-Jianpi decoction; IL, Interleukin; IFN-γ, Interferon-γ; TACE, Transcatheter arterial chemoembolization.
