# Supplementary material for: Network Pharmacology-Based Study on the Mechanism of Bushen-Jianpi Decoction in Liver Cancer Treatment
Source: Evid Based Complement Alternat Med. 2019 Mar 19;2019:3242989. doi: 10.1155/2019/3242989 (PMC6444272; doi:10.1155/2019/3242989)
Supplement: Supplementary 3 — Supplementary Table 2: all ingredients and targets of BSJPD. [file 3242989.f3.pdf]

Supplementary Table 2. All ingredients and targets of BSJPD. BSJPD, Bushen-Jianpi decoction.

**Chemical ingredients of BSJPD**	**Targets of BSJPD**
kaempferol	PSMD3
quercetin	PSMD3
formononetin	HSD3B1
naringenin	HMGCR
Isofucosterol	ABAT
naringenin	ABAT
beta-sitosterol	HTR2A
Stigmasterol	HTR2A
Tetrahydroalstonine	HTR2A
Fumarine	HTR2A
Medicarpin	HTR2A
Vestitol	HTR2A
Inermin	HTR3A
Fumarine	HTR3A
Inermine	HTR3A
shinpterocarpin	HTR3A
quercetin	MMP2
quercetin	HSPA5
Kadsurenone	ACHE
Tetrahydroalstonine	ACHE
kaempferol	ACHE
quercetin	ACHE
Frutinone A	ACHE
3β-acetoxyatractylone	ACHE
isorhamnetin	ACHE
7-Methoxy-2-methyl isoflavone	ACHE
formononetin	ACHE
glyasperin B	ACHE
Glyasperin C	ACHE
(2S)-6-(2,4-dihydroxyphenyl)-2-(2-hydroxypropan-2-yl)-4-methoxy-2	ACHE
Semilicoisoflavone B	ACHE
Phaseolinisoflavan	ACHE
Gancaonin A	ACHE
Licoisoflavone B	ACHE
licoisoflavanone	ACHE
licopyranocoumarin	ACHE
Glabridin	ACHE
Glabrone	ACHE
(-)-Medicocarpin	ACHE
3'-Methoxyglabridin	ACHE
2-[(3R)-8,8-dimethyl-3,4-dihydro-2H-pyrano[6,5-f]chromen-3-yl]-5-r	ACHE
7-Acetoxy-2-methylisoflavone	ACHE
Licoagrocarpin	ACHE
Glyasperins M	ACHE
Glycyrrhiza flavonol A	ACHE
quercetin	ACACA
kaempferol	AHSA1
quercetin	AHSA1
naringenin	ADIPOQ
Isofucosterol	ADH1A
Isofucosterol	ADH1B
hederagenin	ADH1B
Stigmasterol	ADH1C
Isofucosterol	ADH1C
hederagenin	ADH1C
naringenin	AKR1C1
kaempferol	AKR1C3
Stigmasterol	AKR1B1
quercetin	AKR1B1
isorhamnetin	AKR1B1
Stigmasterol	ADRA1A
beta-sitosterol	ADRA1A
3β-acetoxyatractylone	ADRA1A
Medicarpin	ADRA1A
formononetin	ADRA1A
Vestitol	ADRA1A
Stigmasterol	ADRA1B
piperlonguminine	ADRA1B
Kadsurenone	ADRA1B
hancinone C	ADRA1B
beta-sitosterol	ADRA1B

Tetrahydroalstonine	ADRA1B
kaempferol	ADRA1B
hederagenin	ADRA1B
Inermin	ADRA1B
Fumarine	ADRA1B
Inermine	ADRA1B
Medicarpin	ADRA1B
7-Methoxy-2-methyl isoflavone	ADRA1B
(E)-1-(2,4-dihydroxyphenyl)-3-(2,2-dimethylchromen-6-yl)prop-2-en	ADRA1B
Glepidotin B	ADRA1B
Phaseolinisoflavan	ADRA1B
Glypallichalcone	ADRA1B
Gancaonin B	ADRA1B
shinpterocarpin	ADRA1B
Glabridin	ADRA1B
(2S)-7-hydroxy-2-(4-hydroxyphenyl)-8-(3-methylbut-2-enyl)chroman	ADRA1B
1-Methoxyphaseollidin	ADRA1B
3'-Hydroxy-4'-O-Methylglabridin	ADRA1B
licochalcone a	TP53
licochalcone a	ADRA1B
3'-Methoxyglabridin	ADRA1B
2-[(3R)-8,8-dimethyl-3,4-dihydro-2H-pyrano[6,5-f]chromen-3-yl]-5-r	ADRA1B
7-Acetoxy-2-methylisoflavone	ADRA1B
Vestitol	ADRA1B
Licoagrocarpin	ADRA1B
Kadsurenone	ADRA1D
hancinone C	ADRA1D
Tetrahydroalstonine	ADRA1D
Inermin	ADRA1D
suchilactone	ADRA1D
Fumarine	ADRA1D
Inermine	ADRA1D
Medicarpin	ADRA1D
7-Methoxy-2-methyl isoflavone	ADRA1D
shinpterocarpin	ADRA1D
1-Methoxyphaseollidin	ADRA1D
7-Acetoxy-2-methylisoflavone	ADRA1D
Stigmasterol	ADRA2A
Tetrahydroalstonine	ADRA2C
Stigmasterol	MAOA
Stigmasterol	MAOA
Stigmasterol	MAOB
piperlonguminine	MAOB
quercetin	MAOB
DFV	MAOB
isorhamnetin	MAOB
7-Methoxy-2-methyl isoflavone	MAOB
formononetin	MAOB
Glypallichalcone	MAOB
(2R)-7-hydroxy-2-(4-hydroxyphenyl)chroman-4-one	MAOB
HMO	MAOB
AIDS180907	AR
Tetrahydroalstonine	AR
kaempferol	AR
quercetin	AR
Deoxyharringtonine	AR
Frutinone A	AR
3β-acetoxyatractylone	AR
Jaranol	AR
isorhamnetin	AR
Lupiwighteone	AR
7-Methoxy-2-methyl isoflavone	AR
formononetin	AR
Calycosin	AR
(2S)-2-[4-hydroxy-3-(3-methylbut-2-enyl)phenyl]-8,8-dimethyl-2,3-d	AR
glyasperin B	AR
glyasperin F	AR
Glyasperin C	AR
Isotrifoliol	AR
(E)-1-(2,4-dihydroxyphenyl)-3-(2,2-dimethylchromen-6-yl)prop-2-en	AR
kanzonols W	AR
(2S)-6-(2,4-dihydroxyphenyl)-2-(2-hydroxypropan-2-yl)-4-methoxy-2	AR
Semilicoisoflavone B	AR

Glepidotin A	AR
Phaseolinisoflavan	AR
Glypallichalcone	AR
Licochalcone B	AR
licochalcone G	AR
3-(2,4-dihydroxyphenyl)-8-(1,1-dimethylprop-2-enyl)-7-hydroxy-5-m	AR
Licoricone	AR
Gancaonin A	AR
Gancaonin B	AR
3-(3,4-dihydroxyphenyl)-5,7-dihydroxy-8-(3-methylbut-2-enyl)chrom	AR
5,7-dihydroxy-3-(4-methoxyphenyl)-8-(3-methylbut-2-enyl)chromone	AR
2-(3,4-dihydroxyphenyl)-5,7-dihydroxy-6-(3-methylbut-2-enyl)chrom	AR
Glycyrin	AR
Licocoumarone	AR
Licoisoflavone	AR
Licoisoflavone B	AR
licoisoflavanone	AR
shinpterocarpin	AR
(E)-3-[3,4-dihydroxy-5-(3-methylbut-2-enyl)phenyl]-1-(2,4-dihydrox	AR
licopyranocoumarin	AR
Glyzaglabrin	AR
Glabridin	AR
Glabrene	AR
Glabrone	AR
1,3-dihydroxy-8,9-dimethoxy-6-benzofurano[3,2-c]chromenone	AR
Eurycarpin A	AR
Isoglycyrol	AR
Isolicoflavonol	AR
HMO	AR
1-Methoxyphaseollidin	AR
Quercetin der.	AR
3'-Hydroxy-4'-O-Methylglabridin	AR
licochalcone a	AR
3'-Methoxyglabridin	AR
2-[(3R)-8,8-dimethyl-3,4-dihydro-2H-pyrano[6,5-f]chromen-3-yl]-5-1	AR
Inflacoumarin A	AR
Kanzonol F	AR
7,2',4'-trihydroxy－5-methoxy-3－arylcoumarin	AR
7-Acetoxy-2-methylisoflavone	AR
Vestitol	AR
Gancaonin G	AR
Gancaonin H	AR
Licoagrocarpin	AR
Glyasperins M	AR
Glycyrrhiza flavonol A	AR
Licoagroisoflavone	AR
Odoratin	AR
Phaseol	AR
dehydroglyasperins C	AR
kaempferol	SLPI
naringenin	APOB
beta-sitosterol	BAX
kaempferol	BAX
quercetin	BAX
ginsenoside rh2	BAX
beta-sitosterol	BCL2
kaempferol	BCL2
quercetin	BCL2
naringenin	BCL2
licochalcone a	BCL2
kaempferol	ALOX5
quercetin	ALOX5
kaempferol	AHR
quercetin	AHR
naringenin	GOT1
formononetin	ATP5B
quercetin	ABCG2
quercetin	BIRC5
naringenin	BAD
quercetin	BCL2L1
Stigmasterol	Adrb1
7-Methoxy-2-methyl isoflavone	Adrb1
Stigmasterol	ADRB2

piperlonguminine	ADRB2
Kadsurenone	ADRB2
Diop	ADRB2
beta-sitosterol	ADRB2
Tetrahydroalstonine	ADRB2
quercetin	ADRB2
Inermin	ADRB2
Aposiopolamine	ADRB2
Frutinone A	ADRB2
Girinimbin	ADRB2
suchilactone	ADRB2
Fumarine	ADRB2
3β-acetoxyatractylone	ADRB2
Inermine	ADRB2
DFV	ADRB2
Medicarpin	ADRB2
7-Methoxy-2-methyl isoflavone	ADRB2
formononetin	ADRB2
Calycosin	ADRB2
Phaseolinisoflavan	ADRB2
Glypallichalcone	ADRB2
Licochalcone B	ADRB2
Gancaonin B	ADRB2
2-(3,4-dihydroxyphenyl)-5,7-dihydroxy-6-(3-methylbut-2-enyl)chrom	ADRB2
shinpterocarpin	ADRB2
Glabridin	ADRB2
Glabrene	ADRB2
(2R)-7-hydroxy-2-(4-hydroxyphenyl)chroman-4-one	ADRB2
(2S)-7-hydroxy-2-(4-hydroxyphenyl)-8-(3-methylbut-2-enyl)chroman	ADRB2
HMO	ADRB2
1-Methoxyphaseollidin	ADRB2
3'-Hydroxy-4'-O-Methylglabridin	ADRB2
licochalcone a	ADRB2
3'-Methoxyglabridin	ADRB2
2-[(3R)-8,8-dimethyl-3,4-dihydro-2H-pyrano[6,5-f]chromen-3-yl]-5-r	ADRB2
Inflacoumarin A	ADRB2
7-Acetoxy-2-methylisoflavone	ADRB2
Vestitol	ADRB2
Licoagrocarpin	ADRB2
dehydroglyasperins C	ADRB2
Kadsurenone	BACE1
euchrenone	BACE1
Kadsurenone	KCNMA1
hancinone C	KCNMA1
suchilactone	KCNMA1
3'-Hydroxy-4'-O-Methylglabridin	KCNMA1
3'-Methoxyglabridin	KCNMA1
2-[(3R)-8,8-dimethyl-3,4-dihydro-2H-pyrano[6,5-f]chromen-3-yl]-5-r	KCNMA1
Glyasperins M	KCNMA1
Kadsurenone	CALM
hancinone C	CALM
AIDS180907	CALM
Hydroxygenkwanin	CALM
Tetrahydroalstonine	CALM
kaempferol	CALM
(+)-catechin	CALM
Inermin	CALM
suchilactone	CALM
Fumarine	CALM
Inermine	CALM
Jaranol	CALM
Medicarpin	CALM
isorhamnetin	CALM
Lupiwighteone	CALM
7-Methoxy-2-methyl isoflavone	CALM
formononetin	CALM
Calycosin	CALM
(2S)-2-[4-hydroxy-3-(3-methylbut-2-enyl)phenyl]-8,8-dimethyl-2,3-d	CALM
euchrenone	CALM
glyasperin B	CALM
glyasperin F	CALM
Glyasperin C	CALM
(E)-1-(2,4-dihydroxyphenyl)-3-(2,2-dimethylchromen-6-yl)prop-2-en	CALM

kanzonols W	CALM
(2S)-6-(2,4-dihydroxyphenyl)-2-(2-hydroxypropan-2-yl)-4-methoxy-2	CALM
Semilicoisoflavone B	CALM
Glepidotin A	CALM
Glepidotin B	CALM
Phaseolinisoflavan	CALM
Glypallichalcone	CALM
Licochalcone B	CALM
licochalcone G	CALM
3-(2,4-dihydroxyphenyl)-8-(1,1-dimethylprop-2-enyl)-7-hydroxy-5-m	CALM
Licoricone	CALM
Gancaonin A	CALM
Gancaonin B	CALM
3-(3,4-dihydroxyphenyl)-5,7-dihydroxy-8-(3-methylbut-2-enyl)chrom	CALM
5,7-dihydroxy-3-(4-methoxyphenyl)-8-(3-methylbut-2-enyl)chromone	CALM
2-(3,4-dihydroxyphenyl)-5,7-dihydroxy-6-(3-methylbut-2-enyl)chrom	CALM
Glycyrin	CALM
Licoisoflavone	CALM
Licoisoflavone B	CALM
licoisoflavanone	CALM
shinpterocarpin	CALM
(E)-3-[3,4-dihydroxy-5-(3-methylbut-2-enyl)phenyl]-1-(2,4-dihydrox	CALM
liquiritin	CALM
licopyranocoumarin	CALM
Glabridin	CALM
Glabranin	CALM
Glabrene	CALM
Glabrone	CALM
Eurycarpin A	CALM
Sigmoidin-B	CALM
(2R)-7-hydroxy-2-(4-hydroxyphenyl)chroman-4-one	CALM
(2S)-7-hydroxy-2-(4-hydroxyphenyl)-8-(3-methylbut-2-enyl)chroman	CALM
Isolicoflavonol	CALM
HMO	CALM
1-Methoxyphaseollidin	CALM
Quercetin der.	CALM
3'-Hydroxy-4'-O-Methylglabridin	CALM
licochalcone a	CALM
3'-Methoxyglabridin	CALM
2-[(3R)-8,8-dimethyl-3,4-dihydro-2H-pyrano[6,5-f]chromen-3-yl]-5-r	CALM
Inflacoumarin A	CALM
Kanzonol F	CALM
6-prenylated eriodictyol	CALM
7-Acetoxy-2-methylisoflavone	CALM
8-prenylated eriodictyol	CALM
Vestitol	CALM
Gancaonin G	CALM
Gancaonin H	CALM
Licoagrocarpin	CALM
Glyasperins M	CALM
Glycyrrhiza flavonol A	CALM
Licoagroisoflavone	CALM
Odoratin	CALM
Xambioona	CALM
dehydroglyasperins C	CALM
DFV	PKIA
7-Methoxy-2-methyl isoflavone	PKIA
formononetin	PKIA
Glypallichalcone	PKIA
(2R)-7-hydroxy-2-(4-hydroxyphenyl)chroman-4-one	PKIA
HMO	PKIA
Vestitol	PKIA
diosgenin	ABCC2
Kadsurenone	CA2
(E)-1-(2,4-dihydroxyphenyl)-3-(2,2-dimethylchromen-6-yl)prop-2-en	CA2
Glypallichalcone	CA2
Licochalcone B	CA2
licochalcone a	CA2
ginsenoside rh2	CASP1
beta-sitosterol	CASP3
kaempferol	CASP3
quercetin	CASP3
ginsenoside rh2	CASP3

naringenin	CASP3
beta-sitosterol	CASP8
quercetin	CASP8
beta-sitosterol	CASP9
quercetin	CASP9
diosgenin	CAT
(+)-catechin	CAT
naringenin	CAT
quercetin	CTSD
quercetin	CAV1
quercetin	CCL2
quercetin	CD40LG
5-[[5-(4-methoxyphenyl)-2-furyl]methylene]barbituric acid	CDK2
Jaranol	CDK2
Medicarpin	CDK2
isorhamnetin	CDK2
Lupiwighteone	CDK2
7-Methoxy-2-methyl isoflavone	CDK2
formononetin	CDK2
Calycosin	CDK2
glyasperin B	CDK2
glyasperin F	CDK2
Glyasperin C	CDK2
Isotrifoliol	CDK2
(E)-1-(2,4-dihydroxyphenyl)-3-(2,2-dimethylchromen-6-yl)prop-2-en	CDK2
kanzonols W	CDK2
(2S)-6-(2,4-dihydroxyphenyl)-2-(2-hydroxypropan-2-yl)-4-methoxy-2	CDK2
Semilicoisoflavone B	CDK2
Glepidotin A	CDK2
Phaseolinisoflavan	CDK2
Glypallichalcone	CDK2
Licochalcone B	CDK2
licochalcone G	CDK2
3-(2,4-dihydroxyphenyl)-8-(1,1-dimethylprop-2-enyl)-7-hydroxy-5-m	CDK2
3-(3,4-dihydroxyphenyl)-5,7-dihydroxy-8-(3-methylbut-2-enyl)chrom	CDK2
5,7-dihydroxy-3-(4-methoxyphenyl)-8-(3-methylbut-2-enyl)chromone	CDK2
2-(3,4-dihydroxyphenyl)-5,7-dihydroxy-6-(3-methylbut-2-enyl)chrom	CDK2
Licocoumarone	CDK2
Licoisoflavone	CDK2
Licoisoflavone B	CDK2
licoisoflavanone	CDK2
shinpterocarpin	CDK2
(E)-3-[3,4-dihydroxy-5-(3-methylbut-2-enyl)phenyl]-1-(2,4-dihydrox	CDK2
licopyranocoumarin	CDK2
Glyzaglabrin	CDK2
Glabridin	CDK2
Glabrene	CDK2
Glabrone	CDK2
1,3-dihydroxy-9-methoxy-6-benzofurano[3,2-c]chromenone	CDK2
1,3-dihydroxy-8,9-dimethoxy-6-benzofurano[3,2-c]chromenone	CDK2
Eurycarpin A	CDK2
Isolicoflavonol	CDK2
HMO	CDK2
1-Methoxyphaseollidin	CDK2
Quercetin der.	CDK2
3'-Hydroxy-4'-O-Methylglabridin	CDK2
licochalcone a	CDK2
3'-Methoxyglabridin	CDK2
2-[(3R)-8,8-dimethyl-3,4-dihydro-2H-pyrano[6,5-f]chromen-3-yl]-5-r	CDK2
7,2',4'-trihydroxy－5-methoxy-3－arylcoumarin	CDK2
7-Acetoxy-2-methylisoflavone	CDK2
Vestitol	CDK2
Licoagrocarpin	CDK2
Glyasperins M	CDK2
Glycyrrhiza flavonol A	CDK2
Licoagroisoflavone	CDK2
Odoratin	CDK2
Phaseol	CDK2
dehydroglyasperins C	CDK2
licochalcone a	CDK4
diosgenin	TP53
quercetin	TP53
piperlonguminine	PDE3A

Kadsurenone	PDE3A
hancinone C	PDE3A
beta-sitosterol	PDE3A
hederagenin	PDE3A
Frutinone A	PDE3A
suchilactone	PDE3A
Fumarine	PDE3A
Medicarpin	PDE3A
7-Methoxy-2-methyl isoflavone	PDE3A
formononetin	PDE3A
Calycosin	PDE3A
Glepidotin A	PDE3A
Glepidotin B	PDE3A
Glypallichalcone	PDE3A
Licochalcone B	PDE3A
Glabranin	PDE3A
(2R)-7-hydroxy-2-(4-hydroxyphenyl)chroman-4-one	PDE3A
(2S)-7-hydroxy-2-(4-hydroxyphenyl)-8-(3-methylbut-2-enyl)chroman	PDE3A
HMO	PDE3A
7-Acetoxy-2-methylisoflavone	PDE3A
Vestitol	PDE3A
Stigmasterol	CTRB1
quercetin	CLDN4
hancinone C	F7
AIDS180907	F7
kaempferol	F7
quercetin	F7
suchilactone	F7
Fumarine	F7
isorhamnetin	F7
glyasperin B	F7
(2S)-6-(2,4-dihydroxyphenyl)-2-(2-hydroxypropan-2-yl)-4-methoxy-2	F7
Semilicoisoflavone B	F7
Glepidotin A	F7
Glepidotin B	F7
3-(2,4-dihydroxyphenyl)-8-(1,1-dimethylprop-2-enyl)-7-hydroxy-5-m	F7
Gancaonin B	F7
2-(3,4-dihydroxyphenyl)-5,7-dihydroxy-6-(3-methylbut-2-enyl)chrom	F7
licoisoflavanone	F7
liquiritin	F7
licopyranocoumarin	F7
3'-Hydroxy-4'-O-Methylglabridin	F7
3'-Methoxyglabridin	F7
6-prenylated eriodictyol	F7
8-prenylated eriodictyol	F7
Glyasperins M	F7
Glycyrrhiza flavonol A	F7
Kadsurenone	F10
hancinone C	F10
AIDS180907	F10
Tetrahydroalstonine	F10
quercetin	F10
suchilactone	F10
Fumarine	F10
Lupiwighteone	F10
(2S)-2-[4-hydroxy-3-(3-methylbut-2-enyl)phenyl]-8,8-dimethyl-2,3-d	F10
euchrenone	F10
glyasperin B	F10
glyasperin F	F10
Glyasperin C	F10
(E)-1-(2,4-dihydroxyphenyl)-3-(2,2-dimethylchromen-6-yl)prop-2-en	F10
kanzonols W	F10
(2S)-6-(2,4-dihydroxyphenyl)-2-(2-hydroxypropan-2-yl)-4-methoxy-2	F10
Semilicoisoflavone B	F10
Glepidotin A	F10
Glepidotin B	F10
Phaseolinisoflavan	F10
licochalcone G	F10
3-(2,4-dihydroxyphenyl)-8-(1,1-dimethylprop-2-enyl)-7-hydroxy-5-m	F10
Licoricone	F10
Gancaonin A	F10
Gancaonin B	F10
3-(3,4-dihydroxyphenyl)-5,7-dihydroxy-8-(3-methylbut-2-enyl)chrom	F10

5,7-dihydroxy-3-(4-methoxyphenyl)-8-(3-methylbut-2-enyl)chromone	F10
2-(3,4-dihydroxyphenyl)-5,7-dihydroxy-6-(3-methylbut-2-enyl)chrom	F10
Glycyrin	F10
Licoisoflavone	F10
Licoisoflavone B	F10
licoisoflavanone	F10
liquiritin	F10
licopyranocoumarin	F10
Glabranin	F10
Glabrene	F10
Glabrone	F10
Eurycarpin A	F10
Sigmoidin-B	F10
(2S)-7-hydroxy-2-(4-hydroxyphenyl)-8-(3-methylbut-2-enyl)chroman	F10
Isolicoflavonol	F10
1-Methoxyphaseollidin	F10
3'-Hydroxy-4'-O-Methylglabridin	F10
licochalcone a	F10
3'-Methoxyglabridin	F10
2-[(3R)-8,8-dimethyl-3,4-dihydro-2H-pyrano[6,5-f]chromen-3-yl]-5-r	F10
Inflacoumarin A	F10
Kanzonol F	F10
6-prenylated eriodictyol	F10
8-prenylated eriodictyol	F10
Gancaonin G	F10
Gancaonin H	F10
Licoagrocarpin	F10
Glyasperins M	F10
Glycyrrhiza flavonol A	F10
Licoagroisoflavone	F10
Xambioona	F10
dehydroglyasperins C	F10
quercetin	COL1A1
quercetin	COL3A1
quercetin	CRP
quercetin	CXCL10
quercetin	CXCL11
quercetin	CXCL2
AIDS180907	CCNA2
Glycyrol	CCNA2
Medicarpin	CCNA2
isorhamnetin	CCNA2
Lupiwighteone	CCNA2
7-Methoxy-2-methyl isoflavone	CCNA2
formononetin	CCNA2
Calycosin	CCNA2
glyasperin B	CCNA2
glyasperin F	CCNA2
Glyasperin C	CCNA2
Isotrifoliol	CCNA2
(E)-1-(2,4-dihydroxyphenyl)-3-(2,2-dimethylchromen-6-yl)prop-2-en	CCNA2
kanzonols W	CCNA2
(2S)-6-(2,4-dihydroxyphenyl)-2-(2-hydroxypropan-2-yl)-4-methoxy-2	CCNA2
Glepidotin A	CCNA2
Phaseolinisoflavan	CCNA2
Glypallichalcone	CCNA2
Licochalcone B	CCNA2
licochalcone G	CCNA2
Gancaonin A	CCNA2
Gancaonin B	CCNA2
3-(3,4-dihydroxyphenyl)-5,7-dihydroxy-8-(3-methylbut-2-enyl)chrom	CCNA2
5,7-dihydroxy-3-(4-methoxyphenyl)-8-(3-methylbut-2-enyl)chromone	CCNA2
2-(3,4-dihydroxyphenyl)-5,7-dihydroxy-6-(3-methylbut-2-enyl)chrom	CCNA2
Licocoumarone	CCNA2
Licoisoflavone	CCNA2
Licoisoflavone B	CCNA2
licoisoflavanone	CCNA2
shinpterocarpin	CCNA2
(E)-3-[3,4-dihydroxy-5-(3-methylbut-2-enyl)phenyl]-1-(2,4-dihydrox	CCNA2
licopyranocoumarin	CCNA2
Glyzaglabrin	CCNA2
Glabridin	CCNA2
Glabrone	CCNA2

1,3-dihydroxy-9-methoxy-6-benzofurano[3,2-c]chromenone	CCNA2
Eurycarpin A	CCNA2
Isolicoflavonol	CCNA2
HMO	CCNA2
1-Methoxyphaseollidin	CCNA2
3'-Hydroxy-4'-O-Methylglabridin	CCNA2
licochalcone a	CCNA2
3'-Methoxyglabridin	CCNA2
2-[(3R)-8,8-dimethyl-3,4-dihydro-2H-pyrano[6,5-f]chromen-3-yl]-5-r	CCNA2
Vestitol	CCNA2
Gancaonin G	CCNA2
Gancaonin H	CCNA2
Licoagrocarpin	CCNA2
Glyasperins M	CCNA2
Glycyrrhiza flavonol A	CCNA2
Licoagroisoflavone	CCNA2
Odoratin	CCNA2
Phaseol	CCNA2
dehydroglyasperins C	CCNA2
diosgenin	CDKN1
quercetin	CDKN1
quercetin	CDKN2A
naringenin	CYP19A1
kaempferol	CYP1A1
quercetin	CYP1A1
kaempferol	CYP1A2
quercetin	CYP1A2
kaempferol	CYP1B1
quercetin	CYP1B1
kaempferol	CYP3A4
quercetin	CYP3A4
diosgenin	PLA2G4A
quercetin	DCAF5
Tetrahydroalstonine	OPRD1
Fumarine	OPRD1
Medicarpin	OPRD1
shinpterocarpin	OPRD1
Kadsurenone	DPP4
hancinone C	DPP4
Hydroxygenkwanin	DPP4
Tetrahydroalstonine	DPP4
kaempferol	DPP4
quercetin	DPP4
Aposiopolamine	DPP4
Frutinone A	DPP4
3β-acetoxyatractylone	DPP4
Jaranol	DPP4
Medicarpin	DPP4
isorhamnetin	DPP4
Lupiwighteone	DPP4
7-Methoxy-2-methyl isoflavone	DPP4
formononetin	DPP4
Calycosin	DPP4
glyasperin B	DPP4
Glyasperin C	DPP4
(2S)-6-(2,4-dihydroxyphenyl)-2-(2-hydroxypropan-2-yl)-4-methoxy-2	DPP4
Glepidotin A	DPP4
3-(2,4-dihydroxyphenyl)-8-(1,1-dimethylprop-2-enyl)-7-hydroxy-5-m	DPP4
Gancaonin A	DPP4
Gancaonin B	DPP4
5,7-dihydroxy-3-(4-methoxyphenyl)-8-(3-methylbut-2-enyl)chromone	DPP4
2-(3,4-dihydroxyphenyl)-5,7-dihydroxy-6-(3-methylbut-2-enyl)chrom	DPP4
Glycyrin	DPP4
Licoisoflavone	DPP4
Glyzaglabrin	DPP4
Glabrone	DPP4
Eurycarpin A	DPP4
Isoglycyrol	DPP4
HMO	DPP4
Quercetin der.	DPP4
Inflacoumarin A	DPP4
7,2',4'-trihydroxy－5-methoxy-3－arylcoumarin	DPP4
7-Acetoxy-2-methylisoflavone	DPP4

Vestitol	DPP4
Gancaonin G	DPP4
Glycyrrhiza flavonol A	DPP4
Licoagroisoflavone	DPP4
Odoratin	DPP4
quercetin	TOP1
quercetin	TOP2A
Kadsurenone	TOP2
hancinone C	TOP2
AIDS180907	TOP2
Leucanthoside	TOP2
kaempferol	TOP2
quercetin	TOP2
Fumarine	TOP2
Lupiwighteone	TOP2
glyasperin B	TOP2
glyasperin F	TOP2
Glyasperin C	TOP2
kanzonols W	TOP2
(2S)-6-(2,4-dihydroxyphenyl)-2-(2-hydroxypropan-2-yl)-4-methoxy-2	TOP2
Semilicoisoflavone B	TOP2
Glepidotin A	TOP2
Glepidotin B	TOP2
3-(2,4-dihydroxyphenyl)-8-(1,1-dimethylprop-2-enyl)-7-hydroxy-5-m	TOP2
Licoricone	TOP2
Gancaonin A	TOP2
Gancaonin B	TOP2
5,7-dihydroxy-3-(4-methoxyphenyl)-8-(3-methylbut-2-enyl)chromone	TOP2
Glycyrin	TOP2
Licoisoflavone	TOP2
Licoisoflavone B	TOP2
licoisoflavanone	TOP2
licopyranocoumarin	TOP2
1-Methoxyphaseollidin	TOP2
3'-Hydroxy-4'-O-Methylglabridin	TOP2
3'-Methoxyglabridin	TOP2
Gancaonin G	TOP2
Gancaonin H	TOP2
Glyasperins M	TOP2
Glycyrrhiza flavonol A	TOP2
beta-sitosterol	DRD1
Tetrahydroalstonine	DRD1
Fumarine	DRD1
Medicarpin	DRD1
7-Methoxy-2-methyl isoflavone	DRD1
quercetin	DUOX2
ginsenoside rh2	MAPK4
quercetin	EGFR
kaempferol	SELE
quercetin	SELE
hancinone C	ESR1
AIDS180907	ESR1
(+)-catechin	ESR1
5-[[5-(4-methoxyphenyl)-2-furyl]methylene]barbituric acid	ESR1
DFV	ESR1
Glycyrol	ESR1
Medicarpin	ESR1
isorhamnetin	ESR1
Lupiwighteone	ESR1
7-Methoxy-2-methyl isoflavone	ESR1
formononetin	ESR1
Calycosin	ESR1
naringenin	ESR1
(2S)-2-[4-hydroxy-3-(3-methylbut-2-enyl)phenyl]-8,8-dimethyl-2,3-d	ESR1
euchrenone	ESR1
glyasperin B	ESR1
glyasperin F	ESR1
Glyasperin C	ESR1
Isotrifoliol	ESR1
(E)-1-(2,4-dihydroxyphenyl)-3-(2,2-dimethylchromen-6-yl)prop-2-en	ESR1
kanzonols W	ESR1
(2S)-6-(2,4-dihydroxyphenyl)-2-(2-hydroxypropan-2-yl)-4-methoxy-2	ESR1
Semilicoisoflavone B	ESR1

Glepidotin A	ESR1
Glepidotin B	ESR1
Phaseolinisoflavan	ESR1
Glypallichalcone	ESR1
8-(6-hydroxy-2-benzofuranyl)-2,2-dimethyl-5-chromenol	ESR1
Licochalcone B	ESR1
licochalcone G	ESR1
3-(2,4-dihydroxyphenyl)-8-(1,1-dimethylprop-2-enyl)-7-hydroxy-5-m	ESR1
Licoricone	ESR1
Gancaonin A	ESR1
Gancaonin B	ESR1
3-(3,4-dihydroxyphenyl)-5,7-dihydroxy-8-(3-methylbut-2-enyl)chrom	ESR1
5,7-dihydroxy-3-(4-methoxyphenyl)-8-(3-methylbut-2-enyl)chromone	ESR1
Glycyrin	ESR1
Licocoumarone	ESR1
Licoisoflavone	ESR1
Licoisoflavone B	ESR1
licoisoflavanone	ESR1
shinpterocarpin	ESR1
(E)-3-[3,4-dihydroxy-5-(3-methylbut-2-enyl)phenyl]-1-(2,4-dihydrox	ESR1
licopyranocoumarin	ESR1
Glyzaglabrin	ESR1
Glabridin	ESR1
Glabranin	ESR1
Glabrene	ESR1
Glabrone	ESR1
1,3-dihydroxy-9-methoxy-6-benzofurano[3,2-c]chromenone	ESR1
1,3-dihydroxy-8,9-dimethoxy-6-benzofurano[3,2-c]chromenone	ESR1
Eurycarpin A	ESR1
Sigmoidin-B	ESR1
(2R)-7-hydroxy-2-(4-hydroxyphenyl)chroman-4-one	ESR1
(2S)-7-hydroxy-2-(4-hydroxyphenyl)-8-(3-methylbut-2-enyl)chroman	ESR1
Isoglycyrol	ESR1
Isolicoflavonol	ESR1
HMO	ESR1
1-Methoxyphaseollidin	ESR1
Quercetin der.	ESR1
3'-Hydroxy-4'-O-Methylglabridin	ESR1
licochalcone a	ESR1
3'-Methoxyglabridin	ESR1
2-[(3R)-8,8-dimethyl-3,4-dihydro-2H-pyrano[6,5-f]chromen-3-yl]-5-	ESR1
Inflacoumarin A	ESR1
Kanzonol F	ESR1
6-prenylated eriodictyol	ESR1
7,2',4'-trihydroxy－5-methoxy-3－arylcoumarin	ESR1
7-Acetoxy-2-methylisoflavone	ESR1
8-prenylated eriodictyol	ESR1
Vestitol	ESR1
Gancaonin G	ESR1
Gancaonin H	ESR1
Licoagrocarpin	ESR1
Glyasperins M	ESR1
Glycyrrhiza flavonol A	ESR1
Licoagroisoflavone	ESR1
Odoratin	ESR1
Phaseol	ESR1
Xambioona	ESR1
dehydroglyasperins C	ESR1
hancinone C	ESR2
Jaranol	ESR2
Medicarpin	ESR2
isorhamnetin	ESR2
Lupiwighteone	ESR2
7-Methoxy-2-methyl isoflavone	ESR2
formononetin	ESR2
Calycosin	ESR2
(2S)-2-[4-hydroxy-3-(3-methylbut-2-enyl)phenyl]-8,8-dimethyl-2,3-d	ESR2
euchrenone	ESR2
glyasperin B	ESR2
glyasperin F	ESR2
Glyasperin C	ESR2
Isotrifoliol	ESR2
(E)-1-(2,4-dihydroxyphenyl)-3-(2,2-dimethylchromen-6-yl)prop-2-en	ESR2

kanzonols W	ESR2
(2S)-6-(2,4-dihydroxyphenyl)-2-(2-hydroxypropan-2-yl)-4-methoxy-2	ESR2
Phaseolinisoflavan	ESR2
Glypallichalcone	ESR2
Licochalcone B	ESR2
licochalcone G	ESR2
3-(2,4-dihydroxyphenyl)-8-(1,1-dimethylprop-2-enyl)-7-hydroxy-5-m	ESR2
Gancaonin A	ESR2
Gancaonin B	ESR2
5,7-dihydroxy-3-(4-methoxyphenyl)-8-(3-methylbut-2-enyl)chromone	ESR2
Glycyrin	ESR2
Licocoumarone	ESR2
Licoisoflavone B	ESR2
licoisoflavanone	ESR2
shinpterocarpin	ESR2
Glyzaglabrin	ESR2
Glabridin	ESR2
Glabrene	ESR2
Glabrone	ESR2
1,3-dihydroxy-9-methoxy-6-benzofurano[3,2-c]chromenone	ESR2
Eurycarpin A	ESR2
(2S)-7-hydroxy-2-(4-hydroxyphenyl)-8-(3-methylbut-2-enyl)chroman	ESR2
HMO	ESR2
1-Methoxyphaseollidin	ESR2
Quercetin der.	ESR2
3'-Hydroxy-4'-O-Methylglabridin	ESR2
licochalcone a	ESR2
3'-Methoxyglabridin	ESR2
2-[(3R)-8,8-dimethyl-3,4-dihydro-2H-pyrano[6,5-f]chromen-3-yl]-5-r	ESR2
Kanzonol F	ESR2
7,2',4'-trihydroxy－5-methoxy-3－arylcoumarin	ESR2
Vestitol	ESR2
Gancaonin G	ESR2
Licoagrocarpin	ESR2
Glyasperins M	ESR2
Glycyrrhiza flavonol A	ESR2
Licoagroisoflavone	ESR2
Odoratin	ESR2
Xambioona	ESR2
dehydroglyasperins C	ESR2
quercetin	SULT1E1
quercetin	ELK1
quercetin	EIF6
licochalcone a	EIF6
diosgenin	FASN
naringenin	FASN
licochalcone a	FOSL2
quercetin	CCND1
licochalcone a	CCND1
quercetin	CCNB1
Stigmasterol	GABRA1
Isofucosterol	GABRA1
beta-sitosterol	GABRA1
kaempferol	GABRA1
quercetin	GABRA1
hederagenin	GABRA1
Aposiopolamine	GABRA1
Frutinone A	GABRA1
Girinimbin	GABRA1
3β-acetoxyatractylone	GABRA1
8β-ethoxy atractylenolide Ⅲ	GABRA1
isorhamnetin	GABRA1
7-Methoxy-2-methyl isoflavone	GABRA1
(2R)-7-hydroxy-2-(4-hydroxyphenyl)chroman-4-one	GABRA1
7-Acetoxy-2-methylisoflavone	GABRA1
beta-sitosterol	GABRA2
kaempferol	GABRA2
hederagenin	GABRA2
Stigmasterol	GABRA3
beta-sitosterol	GABRA3
hederagenin	GABRA3
beta-sitosterol	GABRA5
hederagenin	GABRA5

hederagenin	GABRA6
quercetin	GJA1
Dioscoreside C_qt	NR3C1
Telocinobufagin	NR3C1
Alisol B monoacetate	NR3C1
16β-methoxyalisol B monoacetate	NR3C1
alisol C monoacetate	NR3C1
[(1S,3R)-1-[(2R)-3,3-dimethyloxiran-2-yl]-3-[(5R,8S,9S,10S,11S,14]	NR3C1
hederagenin	Gria2
isorhamnetin	Gria2
naringenin	GSR
kaempferol	GSTM1
quercetin	GSTM1
kaempferol	GSTM2
quercetin	GSTM2
kaempferol	GSTP1
quercetin	GSTP1
naringenin	GSTP1
isorhamnetin	PYGM
AIDS180907	GSK3B
5-[[5-(4-methoxyphenyl)-2-furyl]methylene]barbituric acid	GSK3B
Glycyrol	GSK3B
isorhamnetin	GSK3B
Lupiwighteone	GSK3B
7-Methoxy-2-methyl isoflavone	GSK3B
formononetin	GSK3B
Calycosin	GSK3B
(2S)-2-[4-hydroxy-3-(3-methylbut-2-enyl)phenyl]-8,8-dimethyl-2,3-d	GSK3B
glyasperin B	GSK3B
glyasperin F	GSK3B
Glyasperin C	GSK3B
Isotrifoliol	GSK3B
(E)-1-(2,4-dihydroxyphenyl)-3-(2,2-dimethylchromen-6-yl)prop-2-en	GSK3B
kanzonols W	GSK3B
(2S)-6-(2,4-dihydroxyphenyl)-2-(2-hydroxypropan-2-yl)-4-methoxy-2	GSK3B
Semilicoisoflavone B	GSK3B
Glepidotin A	GSK3B
Phaseolinisoflavan	GSK3B
Glypallichalcone	GSK3B
Licochalcone B	GSK3B
licochalcone G	GSK3B
3-(2,4-dihydroxyphenyl)-8-(1,1-dimethylprop-2-enyl)-7-hydroxy-5-m	GSK3B
Gancaonin A	GSK3B
Gancaonin B	GSK3B
3-(3,4-dihydroxyphenyl)-5,7-dihydroxy-8-(3-methylbut-2-enyl)chrom	GSK3B
5,7-dihydroxy-3-(4-methoxyphenyl)-8-(3-methylbut-2-enyl)chromone	GSK3B
Licocoumarone	GSK3B
Licoisoflavone B	GSK3B
licoisoflavanone	GSK3B
shinpterocarpin	GSK3B
(E)-3-[3,4-dihydroxy-5-(3-methylbut-2-enyl)phenyl]-1-(2,4-dihydrox	GSK3B
Glyzaglabrin	GSK3B
Glabridin	GSK3B
Glabrene	GSK3B
Glabrone	GSK3B
1,3-dihydroxy-9-methoxy-6-benzofurano[3,2-c]chromenone	GSK3B
1,3-dihydroxy-8,9-dimethoxy-6-benzofurano[3,2-c]chromenone	GSK3B
Eurycarpin A	GSK3B
Isoglycyrol	GSK3B
Isolicoflavonol	GSK3B
HMO	GSK3B
1-Methoxyphaseollidin	GSK3B
Quercetin der.	GSK3B
3'-Hydroxy-4'-O-Methylglabridin	GSK3B
licochalcone a	GSK3B
3'-Methoxyglabridin	GSK3B
2-[(3R)-8,8-dimethyl-3,4-dihydro-2H-pyrano[6,5-f]chromen-3-yl]-5-r	GSK3B
7,2',4'-trihydroxy－5-methoxy-3－arylcoumarin	GSK3B
7-Acetoxy-2-methylisoflavone	GSK3B
Vestitol	GSK3B
Gancaonin G	GSK3B
Licoagrocarpin	GSK3B
Glyasperins M	GSK3B

Glycyrrhiza flavonol A	GSK3B
Licoagroisoflavone	GSK3B
Odoratin	GSK3B
Phaseol	GSK3B
quercetin	HSF1
quercetin	HSPB1
(-)-taxifolin	HSP90
Kadsurenone	HSP90
hancinone C	HSP90
beta-sitosterol	HSP90
Hydroxygenkwanin	HSP90
Tetrahydroalstonine	HSP90
kaempferol	HSP90
(+)-catechin	HSP90
5-[[5-(4-methoxyphenyl)-2-furyl]methylene]barbituric acid	HSP90
quercetin	HSP90
Inermin	HSP90
Dianthramine	HSP90
Frutinone A	HSP90
suchilactone	HSP90
Fumarine	HSP90
Inermine	HSP90
DFV	HSP90
Jaranol	HSP90
Medicarpin	HSP90
isorhamnetin	HSP90
Lupiwighteone	HSP90
7-Methoxy-2-methyl isoflavone	HSP90
formononetin	HSP90
Calycosin	HSP90
naringenin	HSP90
glyasperin B	HSP90
glyasperin F	HSP90
Glyasperin C	HSP90
Isotrifoliol	HSP90
Semilicoisoflavone B	HSP90
Glepidotin A	HSP90
Glepidotin B	HSP90
Glypallichalcone	HSP90
8-(6-hydroxy-2-benzofuranyl)-2,2-dimethyl-5-chromenol	HSP90
Licochalcone B	HSP90
licochalcone G	HSP90
3-(2,4-dihydroxyphenyl)-8-(1,1-dimethylprop-2-enyl)-7-hydroxy-5-m	HSP90
Gancaonin A	HSP90
Gancaonin B	HSP90
3-(3,4-dihydroxyphenyl)-5,7-dihydroxy-8-(3-methylbut-2-enyl)chrom	HSP90
5,7-dihydroxy-3-(4-methoxyphenyl)-8-(3-methylbut-2-enyl)chromone	HSP90
2-(3,4-dihydroxyphenyl)-5,7-dihydroxy-6-(3-methylbut-2-enyl)chrom	HSP90
Licocoumarone	HSP90
Licoisoflavone	HSP90
licoisoflavanone	HSP90
(E)-3-[3,4-dihydroxy-5-(3-methylbut-2-enyl)phenyl]-1-(2,4-dihydrox	HSP90
Glyzaglabrin	HSP90
Glabranin	HSP90
Glabrene	HSP90
1,3-dihydroxy-9-methoxy-6-benzofurano[3,2-c]chromenone	HSP90
1,3-dihydroxy-8,9-dimethoxy-6-benzofurano[3,2-c]chromenone	HSP90
Eurycarpin A	HSP90
Sigmoidin-B	HSP90
(2R)-7-hydroxy-2-(4-hydroxyphenyl)chroman-4-one	HSP90
(2S)-7-hydroxy-2-(4-hydroxyphenyl)-8-(3-methylbut-2-enyl)chroman	HSP90
Isolicoflavonol	HSP90
1-Methoxyphaseollidin	HSP90
Quercetin der.	HSP90
3'-Hydroxy-4'-O-Methylglabridin	HSP90
licochalcone a	HSP90
3'-Methoxyglabridin	HSP90
Inflacoumarin A	HSP90
6-prenylated eriodictyol	HSP90
7,2',4'-trihydroxy—5-methoxy-3—arylcoumarin	HSP90
7-Acetoxy-2-methylisoflavone	HSP90
8-prenylated eriodictyol	HSP90
Vestitol	HSP90

Gancaonin G	HSP90
Gancaonin H	HSP90
Licoagrocarpin	HSP90
Glyasperins M	HSP90
Glycyrrhiza flavonol A	HSP90
Odoratin	HSP90
Phaseol	HSP90
dehydroglyasperins C	HSP90
kaempferol	HMOX1
quercetin	HMOX1
quercetin	HK2
quercetin	NKX3-1
kaempferol	HAS2
(+)-catechin	HAS2
quercetin	HAS2
diosgenin	HIF1A
quercetin	HIF1A
Stigmasterol	IGHG1
hederagenin	IGHG1
Inermin	IGHG1
Inermine	IGHG1
7-Methoxy-2-methyl isoflavone	IGHG1
Glepidotin A	IGHG1
Glepidotin B	IGHG1
licochalcone G	IGHG1
Glabridin	IGHG1
HMO	IGHG1
quercetin	IKBKA
kaempferol	IKBKB
kaempferol	INSR
quercetin	INSR
quercetin	IGF2
quercetin	IGFBP3
kaempferol	ICAM1
quercetin	ICAM1
quercetin	IFNG
ginsenoside rh2	IFNG
quercetin	IRF1
quercetin	IL1A
quercetin	IL1B
ginsenoside rh2	IL1B
quercetin	IL10
quercetin	IL2
formononetin	IL4
quercetin	IL6
quercetin	IL8
kaempferol	MMP1
quercetin	MMP1
Stigmasterol	LTA4H
piperlonguminine	LTA4H
7-Methoxy-2-methyl isoflavone	LTA4H
Glypallichalcone	LTA4H
naringenin	CES1
naringenin	LDLR
Isofucosterol	LYZ
hederagenin	LYZ
quercetin	MGAM
quercetin	MMP9
naringenin	MTTP
beta-sitosterol	MAPK2
sitosterol	NR3C2
Stigmasterol	NR3C2
Isofucosterol	NR3C2
diosgenin	NR3C2
CLR	NR3C2
Telocinobufagin	NR3C2
alisol B	NR3C2
(2R)-2-[(3S,5R,10S,13R,14R,16R,17R)-3,16-dihydroxy-4,4,10,13,14	NR3C2
trametenolic acid	NR3C2
Cerevisterol	NR3C2
Deoxyharringtonine	NR3C2
Ginsenoside-Rh4_qt	NR3C2
quercetin	MAPK1

naringenin	MAPK1
licochalcone a	MAPK1
Medicarpin	MAPK10
Glycyrol	MAPK14
isorhamnetin	MAPK14
Lupiwighteone	MAPK14
7-Methoxy-2-methyl isoflavone	MAPK14
formononetin	MAPK14
Calycosin	MAPK14
(2S)-2-[4-hydroxy-3-(3-methylbut-2-enyl)phenyl]-8,8-dimethyl-2,3-d	MAPK14
glyasperin F	MAPK14
Glyasperin C	MAPK14
Isotrifoliol	MAPK14
(E)-1-(2,4-dihydroxyphenyl)-3-(2,2-dimethylchromen-6-yl)prop-2-en	MAPK14
kanzonols W	MAPK14
(2S)-6-(2,4-dihydroxyphenyl)-2-(2-hydroxypropan-2-yl)-4-methoxy-2	MAPK14
Glepidotin A	MAPK14
Phaseolinisoflavan	MAPK14
Glypallichalcone	MAPK14
Licochalcone B	MAPK14
licochalcone G	MAPK14
3-(2,4-dihydroxyphenyl)-8-(1,1-dimethylprop-2-enyl)-7-hydroxy-5-m	MAPK14
3-(3,4-dihydroxyphenyl)-5,7-dihydroxy-8-(3-methylbut-2-enyl)chrom	MAPK14
5,7-dihydroxy-3-(4-methoxyphenyl)-8-(3-methylbut-2-enyl)chromone	MAPK14
Licoisoflavone	MAPK14
shinpterocarpin	MAPK14
(E)-3-[3,4-dihydroxy-5-(3-methylbut-2-enyl)phenyl]-1-(2,4-dihydrox	MAPK14
Glyzaglabrin	MAPK14
Glabridin	MAPK14
Glabrene	MAPK14
Glabrone	MAPK14
1,3-dihydroxy-9-methoxy-6-benzofurano[3,2-c]chromenone	MAPK14
1,3-dihydroxy-8,9-dimethoxy-6-benzofurano[3,2-c]chromenone	MAPK14
Eurycarpin A	MAPK14
HMO	MAPK14
1-Methoxyphaseollidin	MAPK14
Quercetin der.	MAPK14
3'-Hydroxy-4'-O-Methylglabridin	MAPK14
licochalcone a	MAPK14
3'-Methoxyglabridin	MAPK14
2-[(3R)-8,8-dimethyl-3,4-dihydro-2H-pyrano[6,5-f]chromen-3-yl]-5-r	MAPK14
7,2',4'-trihydroxy－5-methoxy-3－arylcoumarin	MAPK14
7-Acetoxy-2-methylisoflavone	MAPK14
Vestitol	MAPK14
Gancaonin G	MAPK14
Licoagrocarpin	MAPK14
Licoagroisoflavone	MAPK14
Odoratin	MAPK14
Phaseol	MAPK14
dehydroglyasperins C	MAPK14
naringenin	MAPK3
kaempferol	MAPK8
Stigmasterol	PRKACA
beta-sitosterol	PRKACA
Hydroxygenkwanin	PRKACA
Tetrahydroalstonine	PRKACA
kaempferol	PRKACA
(+)-catechin	PRKACA
5-[[5-(4-methoxyphenyl)-2-furyl]methylene]barbituric acid	PRKACA
quercetin	PRKACA
Inermin	PRKACA
Frutinone A	PRKACA
Girinimbin	PRKACA
suchilactone	PRKACA
Fumarine	PRKACA
Inermine	PRKACA
DFV	PRKACA
Medicarpin	PRKACA
isorhamnetin	PRKACA
7-Methoxy-2-methyl isoflavone	PRKACA
formononetin	PRKACA
Calycosin	PRKACA
naringenin	PRKACA

Isotrifoliol	PRKACA
Glypallichalcone	PRKACA
Licochalcone B	PRKACA
shinpterocarpin	PRKACA
Glyzaglabrin	PRKACA
Glabridin	PRKACA
Glabranin	PRKACA
1,3-dihydroxy-9-methoxy-6-benzofurano[3,2-c]chromenone	PRKACA
1,3-dihydroxy-8,9-dimethoxy-6-benzofurano[3,2-c]chromenone	PRKACA
(2R)-7-hydroxy-2-(4-hydroxyphenyl)chroman-4-one	PRKACA
HMO	PRKACA
3'-Hydroxy-4'-O-Methylglabridin	PRKACA
2-[(3R)-8,8-dimethyl-3,4-dihydro-2H-pyrano[6,5-f]chromen-3-yl]-5-r	PRKACA
7,2',4'-trihydroxy－5-methoxy-3－arylcoumarin	PRKACA
Vestitol	PRKACA
Glyasperins M	PRKACA
Phaseol	PRKACA
Leucanthoside	PTPN1
suchilactone	PTPN1
isorhamnetin	PTPN1
3-(3,4-dihydroxyphenyl)-5,7-dihydroxy-8-(3-methylbut-2-enyl)chrom	PTPN1
Quercetin der.	PTPN1
naringenin	ABCC1
Stigmasterol	CHRM1
piperlonguminine	CHRM1
Kadsurenone	CHRM1
hancinone C	CHRM1
beta-sitosterol	CHRM1
Tetrahydroalstonine	CHRM1
kaempferol	CHRM1
hederagenin	CHRM1
Aposiopolamine	CHRM1
Fumarine	CHRM1
3β-acetoxyatractylone	CHRM1
Inermine	CHRM1
Medicarpin	CHRM1
7-Methoxy-2-methyl isoflavone	CHRM1
formononetin	CHRM1
Phaseolinisoflavan	CHRM1
Glypallichalcone	CHRM1
shinpterocarpin	CHRM1
Glabridin	CHRM1
HMO	CHRM1
licochalcone a	CHRM1
2-[(3R)-8,8-dimethyl-3,4-dihydro-2H-pyrano[6,5-f]chromen-3-yl]-5-r	CHRM1
Vestitol	CHRM1
Licoagrocarpin	CHRM1
Stigmasterol	CHRM2
Kadsurenone	CHRM2
beta-sitosterol	CHRM2
kaempferol	CHRM2
hederagenin	CHRM2
3β-acetoxyatractylone	CHRM2
Medicarpin	CHRM2
Stigmasterol	CHRM3
Kadsurenone	CHRM3
hancinone C	CHRM3
Diop	CHRM3
beta-sitosterol	CHRM3
Tetrahydroalstonine	CHRM3
hederagenin	CHRM3
Inermin	CHRM3
Aposiopolamine	CHRM3
Fumarine	CHRM3
3β-acetoxyatractylone	CHRM3
Inermine	CHRM3
Medicarpin	CHRM3
7-Methoxy-2-methyl isoflavone	CHRM3
shinpterocarpin	CHRM3
2-[(3R)-8,8-dimethyl-3,4-dihydro-2H-pyrano[6,5-f]chromen-3-yl]-5-r	CHRM3
Licoagrocarpin	CHRM3
beta-sitosterol	CHRM4
Tetrahydroalstonine	CHRM4

Fumarine	CHRM4
Medicarpin	CHRM4
Vestitol	CHRM4
Kadsurenone	CHRM5
Tetrahydroalstonine	CHRM5
Fumarine	CHRM5
Medicarpin	CHRM5
7-Methoxy-2-methyl isoflavone	CHRM5
Licoagrocarpin	CHRM5
Kadsurenone	OPRM1
beta-sitosterol	OPRM1
Tetrahydroalstonine	OPRM1
Fumarine	OPRM1
3β-acetoxyatractylone	OPRM1
Inermine	OPRM1
Medicarpin	OPRM1
7-Methoxy-2-methyl isoflavone	OPRM1
shinpterocarpin	OPRM1
quercetin	MYC
quercetin	MPO
quercetin	NQO1
formononetin	SIRT1
formononetin	MT-ND6
quercetin	POR
Stigmasterol	CHRNA7
beta-sitosterol	CHRNA7
Inermin	CHRNA7
Frutinone A	CHRNA7
Girinimbin	CHRNA7
3β-acetoxyatractylone	CHRNA7
8β-ethoxy atractylenolide Ⅲ	CHRNA7
Medicarpin	CHRNA7
7-Methoxy-2-methyl isoflavone	CHRNA7
shinpterocarpin	CHRNA7
beta-sitosterol	CHRNA2
quercetin	NCF1
isorhamnetin	NCF1
quercetin	NFKBIA
ginsenoside rh2	NFKBIA
quercetin	NOS3
hancinone C	NOS2
AIDS180907	NOS2
Hydroxygenkwanin	NOS2
Tetrahydroalstonine	NOS2
kaempferol	NOS2
Glycyrol	NOS2
Jaranol	NOS2
Medicarpin	NOS2
isorhamnetin	NOS2
Lupiwighteone	NOS2
7-Methoxy-2-methyl isoflavone	NOS2
formononetin	NOS2
Calycosin	NOS2
(2S)-2-[4-hydroxy-3-(3-methylbut-2-enyl)phenyl]-8,8-dimethyl-2,3-d	NOS2
euchrenone	NOS2
glyasperin B	NOS2
glyasperin F	NOS2
Glyasperin C	NOS2
Isotrifoliol	NOS2
(E)-1-(2,4-dihydroxyphenyl)-3-(2,2-dimethylchromen-6-yl)prop-2-en	NOS2
kanzonols W	NOS2
(2S)-6-(2,4-dihydroxyphenyl)-2-(2-hydroxypropan-2-yl)-4-methoxy-2	NOS2
Semilicoisoflavone B	NOS2
Glepidotin A	NOS2
Phaseolinisoflavan	NOS2
Glypallichalcone	NOS2
8-(6-hydroxy-2-benzofuranyl)-2,2-dimethyl-5-chromenol	NOS2
Licochalcone B	NOS2
licochalcone G	NOS2
3-(2,4-dihydroxyphenyl)-8-(1,1-dimethylprop-2-enyl)-7-hydroxy-5-m	NOS2
Licoricone	NOS2
Gancaonin A	NOS2
Gancaonin B	NOS2

3-(3,4-dihydroxyphenyl)-5,7-dihydroxy-8-(3-methylbut-2-enyl)chrom	NOS2
5,7-dihydroxy-3-(4-methoxyphenyl)-8-(3-methylbut-2-enyl)chromone	NOS2
Glycyrin	NOS2
Licoisoflavone	NOS2
Licoisoflavone B	NOS2
licoisoflavanone	NOS2
shinpterocarpin	NOS2
licopyranocoumarin	NOS2
Glyzaglabrin	NOS2
Glabridin	NOS2
Glabranin	NOS2
Glabrene	NOS2
Glabrone	NOS2
Eurycarpin A	NOS2
(2S)-7-hydroxy-2-(4-hydroxyphenyl)-8-(3-methylbut-2-enyl)chroman	NOS2
Isoglycyrol	NOS2
Isolicoflavonol	NOS2
HMO	NOS2
1-Methoxyphaseollidin	NOS2
Quercetin der.	NOS2
3'-Hydroxy-4'-O-Methylglabridin	NOS2
licochalcone a	NOS2
3'-Methoxyglabridin	NOS2
2-[(3R)-8,8-dimethyl-3,4-dihydro-2H-pyrano[6,5-f]chromen-3-yl]-5-	NOS2
6-prenylated eriodictyol	NOS2
7,2',4'-trihydroxy－5-methoxy-3－arylcoumarin	NOS2
7-Acetoxy-2-methylisoflavone	NOS2
Vestitol	NOS2
Gancaonin G	NOS2
Licoagrocarpin	NOS2
Glyasperins M	NOS2
Glycyrrhiza flavonol A	NOS2
Licoagroisoflavone	NOS2
Odoratin	NOS2
Xambioona	NOS2
dehydroglyasperins C	NOS2
piperlonguminine	NOS3
kaempferol	NOS3
3β-acetoxyatractylone	NOS3
isorhamnetin	NOS3
7-Methoxy-2-methyl isoflavone	NOS3
formononetin	NOS3
Glepidotin A	NOS3
Glepidotin B	NOS3
Glabranin	NOS3
1-Methoxyphaseollidin	NOS3
2-[(3R)-8,8-dimethyl-3,4-dihydro-2H-pyrano[6,5-f]chromen-3-yl]-5-	NOS3
7-Acetoxy-2-methylisoflavone	NOS3
Gancaonin G	NOS3
Licoagrocarpin	NOS3
quercetin	NFE2L2
Stigmasterol	NCOA1
Kadsurenone	NCOA1
hancinone C	NCOA1
AIDS180907	NCOA1
Inermin	NCOA1
suchilactone	NCOA1
8β-ethoxy atractylenolide Ⅲ	NCOA1
isorhamnetin	NCOA1
7-Methoxy-2-methyl isoflavone	NCOA1
kanzonols W	NCOA1
Glepidotin B	NCOA1
Phaseolinisoflavan	NCOA1
Glypallichalcone	NCOA1
3-(2,4-dihydroxyphenyl)-8-(1,1-dimethylprop-2-enyl)-7-hydroxy-5-m	NCOA1
licoisoflavanone	NCOA1
shinpterocarpin	NCOA1
Glabridin	NCOA1
1-Methoxyphaseollidin	NCOA1
3'-Hydroxy-4'-O-Methylglabridin	NCOA1
3'-Methoxyglabridin	NCOA1
2-[(3R)-8,8-dimethyl-3,4-dihydro-2H-pyrano[6,5-f]chromen-3-yl]-5-	NCOA1
8-prenylated eriodictyol	NCOA1

Glyasperins M	NCOA1
sitosterol	NCOA2
Stigmasterol	NCOA2
Kadsurenone	NCOA2
hancinone C	NCOA2
Isofucosterol	NCOA2
Dioscoreside C_qt	NCOA2
AIDS180907	NCOA2
CLR	NCOA2
Mandenol	NCOA2
Ethyl linolenate	NCOA2
poriferast-5-en-3beta-ol	NCOA2
Ethyl oleate (NF)	NCOA2
beta-sitosterol	NCOA2
Cornudentanone	NCOA2
Hydroxygenkwanin	NCOA2
kaempferol	NCOA2
(+)-catechin	NCOA2
quercetin	NCOA2
(2R)-2-[(3S,5R,10S,13R,14R,16R,17R)-3,16-dihydroxy-4,4,10,13,14	NCOA2
hederagenin	NCOA2
arachidonate	NCOA2
Ginsenoside-Rh4_qt	NCOA2
Girinimbin	NCOA2
8β-ethoxy atractylenolide Ⅲ	NCOA2
Jaranol	NCOA2
isorhamnetin	NCOA2
Lupiwighteone	NCOA2
7-Methoxy-2-methyl isoflavone	NCOA2
Calycosin	NCOA2
glyasperin B	NCOA2
Glyasperin C	NCOA2
(E)-1-(2,4-dihydroxyphenyl)-3-(2,2-dimethylchromen-6-yl)prop-2-en	NCOA2
kanzonols W	NCOA2
licochalcone G	NCOA2
3-(2,4-dihydroxyphenyl)-8-(1,1-dimethylprop-2-enyl)-7-hydroxy-5-m	NCOA2
Licoricone	NCOA2
Gancaonin A	NCOA2
Gancaonin B	NCOA2
3-(3,4-dihydroxyphenyl)-5,7-dihydroxy-8-(3-methylbut-2-enyl)chrom	NCOA2
5,7-dihydroxy-3-(4-methoxyphenyl)-8-(3-methylbut-2-enyl)chromone	NCOA2
Glycyrin	NCOA2
Licoisoflavone	NCOA2
(E)-3-[3,4-dihydroxy-5-(3-methylbut-2-enyl)phenyl]-1-(2,4-dihydrox	NCOA2
Glabridin	NCOA2
Glabrene	NCOA2
Isolicoflavonol	NCOA2
1-Methoxyphaseollidin	NCOA2
Quercetin der.	NCOA2
3'-Hydroxy-4'-O-Methylglabridin	NCOA2
licochalcone a	NCOA2
3'-Methoxyglabridin	NCOA2
2-[(3R)-8,8-dimethyl-3,4-dihydro-2H-pyrano[6,5-f]chromen-3-yl]-5-r	NCOA2
Inflacoumarin A	NCOA2
icos-5-enoic acid	NCOA2
Kanzonol F	NCOA2
7-Acetoxy-2-methylisoflavone	NCOA2
gadelaidic acid	NCOA2
Gancaonin G	NCOA2
Gancaonin H	NCOA2
Licoagrocarpin	NCOA2
Glyasperins M	NCOA2
Odoratin	NCOA2
Xambioona	NCOA2
dehydroglyasperins C	NCOA2
diosgenin	NR1I2
kaempferol	NR1I2
quercetin	NR1I2
kaempferol	NR1I3
quercetin	NR1I3
quercetin	ODC1
quercetin	SPP1
isorhamnetin	PPARD

Glyasperins M	PPARD
Tetrahydroalstonine	PPARG
kaempferol	PPARG
quercetin	PPARG
Frutinone A	PPARG
Glycyrol	PPARG
isorhamnetin	PPARG
Lupiwighteone	PPARG
7-Methoxy-2-methyl isoflavone	PPARG
formononetin	PPARG
Calycosin	PPARG
(2S)-2-[4-hydroxy-3-(3-methylbut-2-enyl)phenyl]-8,8-dimethyl-2,3-d	PPARG
glyasperin B	PPARG
glyasperin F	PPARG
Glyasperin C	PPARG
(E)-1-(2,4-dihydroxyphenyl)-3-(2,2-dimethylchromen-6-yl)prop-2-en	PPARG
kanzonols W	PPARG
(2S)-6-(2,4-dihydroxyphenyl)-2-(2-hydroxypropan-2-yl)-4-methoxy-2	PPARG
Semilicoisoflavone B	PPARG
Glepidotin A	PPARG
Phaseolinisoflavan	PPARG
Glypallichalcone	PPARG
Licochalcone B	PPARG
licochalcone G	PPARG
3-(2,4-dihydroxyphenyl)-8-(1,1-dimethylprop-2-enyl)-7-hydroxy-5-m	PPARG
Licoricone	PPARG
Gancaonin A	PPARG
Gancaonin B	PPARG
3-(3,4-dihydroxyphenyl)-5,7-dihydroxy-8-(3-methylbut-2-enyl)chrom	PPARG
5,7-dihydroxy-3-(4-methoxyphenyl)-8-(3-methylbut-2-enyl)chromone	PPARG
2-(3,4-dihydroxyphenyl)-5,7-dihydroxy-6-(3-methylbut-2-enyl)chrom	PPARG
Glycyrin	PPARG
Licoisoflavone	PPARG
Licoisoflavone B	PPARG
licoisoflavanone	PPARG
shinpterocarpin	PPARG
(E)-3-[3,4-dihydroxy-5-(3-methylbut-2-enyl)phenyl]-1-(2,4-dihydrox	PPARG
licopyranocoumarin	PPARG
Glyzaglabrin	PPARG
Glabridin	PPARG
Glabrene	PPARG
Glabrone	PPARG
1,3-dihydroxy-9-methoxy-6-benzofurano[3,2-c]chromenone	PPARG
1,3-dihydroxy-8,9-dimethoxy-6-benzofurano[3,2-c]chromenone	PPARG
Eurycarpin A	PPARG
Isolicoflavonol	PPARG
HMO	PPARG
1-Methoxyphaseollidin	PPARG
Quercetin der.	PPARG
3'-Hydroxy-4'-O-Methylglabridin	PPARG
licochalcone a	PPARG
3'-Methoxyglabridin	PPARG
2-[(3R)-8,8-dimethyl-3,4-dihydro-2H-pyrano[6,5-f]chromen-3-yl]-5-1	PPARG
Inflacoumarin A	PPARG
7,2',4'-trihydroxy—5-methoxy-3—arylcoumarin	PPARG
7-Acetoxy-2-methylisoflavone	PPARG
Vestitol	PPARG
Gancaonin G	PPARG
Licoagrocarpin	PPARG
Glyasperins M	PPARG
Licoagroisoflavone	PPARG
Odoratin	PPARG
Phaseol	PPARG
dehydroglyasperins C	PPARG
quercetin	PPARA
naringenin	PPARA
quercetin	PPARD
naringenin	PPARG
quercetin	PTEN
(-)-taxifolin	PIK3CG
beta-sitosterol	PIK3CG
Hydroxygenkwanin	PIK3CG
kaempferol	PIK3CG

quercetin	PIK3CG
Inermin	PIK3CG
Frutinone A	PIK3CG
Girinimbin	PIK3CG
Inermine	PIK3CG
DFV	PIK3CG
Medicarpin	PIK3CG
isorhamnetin	PIK3CG
naringenin	PIK3CG
Isotrifoliol	PIK3CG
8-(6-hydroxy-2-benzofuranyl)-2,2-dimethyl-5-chromenol	PIK3CG
shinpterocarpin	PIK3CG
Glyzaglabrin	PIK3CG
(2R)-7-hydroxy-2-(4-hydroxyphenyl)chroman-4-one	PIK3CG
1-Methoxyphaseollidin	PIK3CG
naringenin	PLB1
ginsenoside rh2	ADCYAP1
quercetin	SERPINE1
quercetin	PARP1
Kadsurenone	KCNH2
hancinone C	KCNH2
beta-sitosterol	KCNH2
Tetrahydroalstonine	KCNH2
quercetin	KCNH2
suchilactone	KCNH2
Fumarine	KCNH2
(2S)-2-[4-hydroxy-3-(3-methylbut-2-enyl)phenyl]-8,8-dimethyl-2,3-d	KCNH2
euchrenone	KCNH2
Glyasperin C	KCNH2
3-(2,4-dihydroxyphenyl)-8-(1,1-dimethylprop-2-enyl)-7-hydroxy-5-m	KCNH2
Licoricone	KCNH2
5,7-dihydroxy-3-(4-methoxyphenyl)-8-(3-methylbut-2-enyl)chromone	KCNH2
Glycyrin	KCNH2
shinpterocarpin	KCNH2
1-Methoxyphaseollidin	KCNH2
3'-Hydroxy-4'-O-Methylglabridin	KCNH2
3'-Methoxyglabridin	KCNH2
2-[(3R)-8,8-dimethyl-3,4-dihydro-2H-pyrano[6,5-f]chromen-3-yl]-5-r	KCNH2
Licoagrocarpin	KCNH2
Glyasperins M	KCNH2
quercetin	PCOLCE
quercetin	EGF
sitosterol	PGR
Stigmasterol	PGR
24-Methylcholest-5-enyl-3belta-O-glucopyranoside_qt	PGR
campesterol	PGR
Isofucosterol	PGR
diosgenin	PGR
CLR	PGR
poriferast-5-en-3beta-ol	PGR
beta-sitosterol	PGR
Mairin	PGR
kaempferol	PGR
ergosta-7,22E-dien-3beta-ol	PGR
Ergosterol peroxide	PGR
hederagenin	PGR
alexandrin_qt	PGR
(3S,8S,9S,10R,13R,14S,17R)-10,13-dimethyl-17-[(2R,5S)-5-propan-	PGR
quercetin	PTGER3
Stigmasterol	PTGS1
(-)-taxifolin	PTGS1
Kadsurenone	PTGS1
Mandenol	PTGS1
Ethyl linolenate	PTGS1
beta-sitosterol	PTGS1
Hydroxygenkwanin	PTGS1
Tetrahydroalstonine	PTGS1
kaempferol	PTGS1
(+)-catechin	PTGS1
quercetin	PTGS1
1-Monolinolein	PTGS1
hederagenin	PTGS1
Inermin	PTGS1

Dianthramine	PTGS1
arachidonate	PTGS1
Frutinone A	PTGS1
Girinimbin	PTGS1
suchilactone	PTGS1
Fumarine	PTGS1
Inermine	PTGS1
DFV	PTGS1
Jaranol	PTGS1
Medicarpin	PTGS1
isorhamnetin	PTGS1
7-Methoxy-2-methyl isoflavone	PTGS1
formononetin	PTGS1
Calycosin	PTGS1
naringenin	PTGS1
glyasperin F	PTGS1
(E)-1-(2,4-dihydroxyphenyl)-3-(2,2-dimethylchromen-6-yl)prop-2-en	PTGS1
kanzonols W	PTGS1
Glepidotin A	PTGS1
Glepidotin B	PTGS1
Glypallichalcone	PTGS1
Licochalcone B	PTGS1
licoisoflavanone	PTGS1
shinpterocarpin	PTGS1
Glyzaglabrin	PTGS1
Glabranin	PTGS1
Glabrene	PTGS1
Glabrone	PTGS1
(2R)-7-hydroxy-2-(4-hydroxyphenyl)chroman-4-one	PTGS1
(2S)-7-hydroxy-2-(4-hydroxyphenyl)-8-(3-methylbut-2-enyl)chroman	PTGS1
HMO	PTGS1
1-Methoxyphaseollidin	PTGS1
Quercetin der.	PTGS1
3'-Hydroxy-4'-O-Methylglabridin	PTGS1
licochalcone a	PTGS1
3'-Methoxyglabridin	PTGS1
2-[(3R)-8,8-dimethyl-3,4-dihydro-2H-pyrano[6,5-f]chromen-3-yl]-5-r	PTGS1
Inflacoumarin A	PTGS1
7,2',4'-trihydroxy－5-methoxy-3－arylcoumarin	PTGS1
7-Acetoxy-2-methylisoflavone	PTGS1
Vestitol	PTGS1
Licoagrocarpin	PTGS1
Glyasperins M	PTGS1
Odoratin	PTGS1
Stigmasterol	PTGS2
(-)-taxifolin	PTGS2
Kadsurenone	PTGS2
hancinone C	PTGS2
diosgenin	PTGS2
AIDS180907	PTGS2
Mandenol	PTGS2
beta-sitosterol	PTGS2
2,6,10,14,18-pentamethylicosa-2,6,10,14,18-pentaene	PTGS2
Cornudentanone	PTGS2
Hydroxygenkwanin	PTGS2
Tetrahydroalstonine	PTGS2
kaempferol	PTGS2
(+)-catechin	PTGS2
quercetin	PTGS2
hederagenin	PTGS2
Inermin	PTGS2
Dianthramine	PTGS2
arachidonate	PTGS2
Frutinone A	PTGS2
ginsenoside rh2	PTGS2
Girinimbin	PTGS2
suchilactone	PTGS2
Fumarine	PTGS2
14-acetyl-12-senecioyl-2E,8Z,10E-atractylentriol	PTGS2
3β-acetoxyatractylone	PTGS2
8β-ethoxy atractylenolide Ⅲ	PTGS2
Inermine	PTGS2
DFV	PTGS2

Glycyrol	PTGS2
Jaranol	PTGS2
Medicarpin	PTGS2
isorhamnetin	PTGS2
Lupiwighteone	PTGS2
7-Methoxy-2-methyl isoflavone	PTGS2
formononetin	PTGS2
Calycosin	PTGS2
naringenin	PTGS2
(2S)-2-[4-hydroxy-3-(3-methylbut-2-enyl)phenyl]-8,8-dimethyl-2,3-d	PTGS2
euchrenone	PTGS2
glyasperin B	PTGS2
glyasperin F	PTGS2
Glyasperin C	PTGS2
Isotrifoliol	PTGS2
(E)-1-(2,4-dihydroxyphenyl)-3-(2,2-dimethylchromen-6-yl)prop-2-en	PTGS2
kanzonols W	PTGS2
(2S)-6-(2,4-dihydroxyphenyl)-2-(2-hydroxypropan-2-yl)-4-methoxy-2	PTGS2
Semilicoisoflavone B	PTGS2
Glepidotin A	PTGS2
Glepidotin B	PTGS2
Phaseolinisoflavan	PTGS2
Glypallichalcone	PTGS2
8-(6-hydroxy-2-benzofuranyl)-2,2-dimethyl-5-chromenol	PTGS2
Licochalcone B	PTGS2
licochalcone G	PTGS2
3-(2,4-dihydroxyphenyl)-8-(1,1-dimethylprop-2-enyl)-7-hydroxy-5-m	PTGS2
Licoricone	PTGS2
Gancaonin A	PTGS2
Gancaonin B	PTGS2
3-(3,4-dihydroxyphenyl)-5,7-dihydroxy-8-(3-methylbut-2-enyl)chrom	PTGS2
5,7-dihydroxy-3-(4-methoxyphenyl)-8-(3-methylbut-2-enyl)chromone	PTGS2
2-(3,4-dihydroxyphenyl)-5,7-dihydroxy-6-(3-methylbut-2-enyl)chrom	PTGS2
Glycyrin	PTGS2
Licoisoflavone	PTGS2
Licoisoflavone B	PTGS2
licoisoflavanone	PTGS2
shinpterocarpin	PTGS2
(E)-3-[3,4-dihydroxy-5-(3-methylbut-2-enyl)phenyl]-1-(2,4-dihydrox	PTGS2
liquiritin	PTGS2
licopyranocoumarin	PTGS2
Glyzaglabrin	PTGS2
Glabridin	PTGS2
Glabranin	PTGS2
Glabrene	PTGS2
Glabrone	PTGS2
Eurycarpin A	PTGS2
(-)-Medicocarpin	PTGS2
Sigmoidin-B	PTGS2
(2R)-7-hydroxy-2-(4-hydroxyphenyl)chroman-4-one	PTGS2
(2S)-7-hydroxy-2-(4-hydroxyphenyl)-8-(3-methylbut-2-enyl)chroman	PTGS2
Isoglycyrol	PTGS2
Isolicoflavonol	PTGS2
HMO	PTGS2
1-Methoxyphaseollidin	PTGS2
Quercetin der.	PTGS2
3'-Hydroxy-4'-O-Methylglabridin	PTGS2
licochalcone a	PTGS2
3'-Methoxyglabridin	PTGS2
2-[(3R)-8,8-dimethyl-3,4-dihydro-2H-pyrano[6,5-f]chromen-3-yl]-5-r	PTGS2
Inflacoumarin A	PTGS2
Kanzonol F	PTGS2
6-prenylated eriodictyol	PTGS2
7,2',4'-trihydroxy－5-methoxy-3－arylcoumarin	PTGS2
7-Acetoxy-2-methylisoflavone	PTGS2
8-prenylated eriodictyol	PTGS2
Vestitol	PTGS2
Gancaonin G	PTGS2
Gancaonin H	PTGS2
Licoagrocarpin	PTGS2
Glyasperins M	PTGS2
Glycyrrhiza flavonol A	PTGS2
Licoagroisoflavone	PTGS2

Odoratin	PTGS2
Phaseol	PTGS2
Xambioona	PTGS2
dehydroglyasperins C	PTGS2
quercetin	ACPP
ginsenoside rh2	PSMG1
quercetin	RUNX1T1
beta-sitosterol	PRKCA
quercetin	PRKCA
quercetin	PRKCB
quercetin	FOS
AIDS180907	PIM1
Leucanthoside	PIM1
Glycyrol	PIM1
Medicarpin	PIM1
isorhamnetin	PIM1
Lupiwighteone	PIM1
7-Methoxy-2-methyl isoflavone	PIM1
formononetin	PIM1
Calycosin	PIM1
(2S)-2-[4-hydroxy-3-(3-methylbut-2-enyl)phenyl]-8,8-dimethyl-2,3-d	PIM1
euchrenone	PIM1
glyasperin B	PIM1
glyasperin F	PIM1
Glyasperin C	PIM1
Isotrifoliol	PIM1
(E)-1-(2,4-dihydroxyphenyl)-3-(2,2-dimethylchromen-6-yl)prop-2-en	PIM1
kanzonols W	PIM1
(2S)-6-(2,4-dihydroxyphenyl)-2-(2-hydroxypropan-2-yl)-4-methoxy-2	PIM1
Glepidotin A	PIM1
Phaseolinisoflavan	PIM1
Licochalcone B	PIM1
licochalcone G	PIM1
3-(2,4-dihydroxyphenyl)-8-(1,1-dimethylprop-2-enyl)-7-hydroxy-5-m	PIM1
Licoricone	PIM1
Gancaonin A	PIM1
Gancaonin B	PIM1
3-(3,4-dihydroxyphenyl)-5,7-dihydroxy-8-(3-methylbut-2-enyl)chrom	PIM1
5,7-dihydroxy-3-(4-methoxyphenyl)-8-(3-methylbut-2-enyl)chromone	PIM1
2-(3,4-dihydroxyphenyl)-5,7-dihydroxy-6-(3-methylbut-2-enyl)chrom	PIM1
Glycyrin	PIM1
Licoisoflavone	PIM1
Licoisoflavone B	PIM1
licoisoflavanone	PIM1
shinpterocarpin	PIM1
(E)-3-[3,4-dihydroxy-5-(3-methylbut-2-enyl)phenyl]-1-(2,4-dihydrox	PIM1
licopyranocoumarin	PIM1
Glyzaglabrin	PIM1
Glabridin	PIM1
Glabrene	PIM1
Glabrone	PIM1
Eurycarpin A	PIM1
Isoglycyrol	PIM1
Isolicoflavonol	PIM1
HMO	PIM1
1-Methoxyphaseollidin	PIM1
3'-Hydroxy-4'-O-Methylglabridin	PIM1
licochalcone a	PIM1
3'-Methoxyglabridin	PIM1
2-[(3R)-8,8-dimethyl-3,4-dihydro-2H-pyrano[6,5-f]chromen-3-yl]-5-1	PIM1
Inflacoumarin A	PIM1
Kanzonol F	PIM1
7,2',4'-trihydroxy－5-methoxy-3－arylcoumarin	PIM1
Vestitol	PIM1
Gancaonin G	PIM1
Gancaonin H	PIM1
Licoagrocarpin	PIM1
Glyasperins M	PIM1
Glycyrrhiza flavonol A	PIM1
Licoagroisoflavone	PIM1
Odoratin	PIM1
Phaseol	PIM1
Xambioona	PIM1

dehydroglyasperins C	PIM1
quercetin	NPEPPS
diosgenin	AKT1
kaempferol	AKT1
quercetin	AKT1
naringenin	AKT1
quercetin	RAF1
quercetin	RASSF1
quercetin	RASA1
quercetin	ERBB2
quercetin	ERBB3
quercetin	RB1
licochalcone a	RB1
Stigmasterol	RXRA
piperlonguminine	RXRA
Kadsurenone	RXRA
(+)-catechin	RXRA
quercetin	RXRA
hederagenin	RXRA
Inermin	RXRA
Frutinone A	RXRA
Girinimbin	RXRA
suchilactone	RXRA
3β-acetoxyatractylone	RXRA
Inermine	RXRA
DFV	RXRA
Medicarpin	RXRA
7-Methoxy-2-methyl isoflavone	RXRA
formononetin	RXRA
Calycosin	RXRA
Glyasperin C	RXRA
(E)-1-(2,4-dihydroxyphenyl)-3-(2,2-dimethylchromen-6-yl)prop-2-en	RXRA
kanzonols W	RXRA
Glepidotin A	RXRA
Glepidotin B	RXRA
Phaseolinisoflavan	RXRA
8-(6-hydroxy-2-benzofuranyl)-2,2-dimethyl-5-chromenol	RXRA
shinpterocarpin	RXRA
Glabridin	RXRA
Glabrene	RXRA
Glabrone	RXRA
(2R)-7-hydroxy-2-(4-hydroxyphenyl)chroman-4-one	RXRA
HMO	RXRA
1-Methoxyphaseollidin	RXRA
3'-Methoxyglabridin	RXRA
2-[(3R)-8,8-dimethyl-3,4-dihydro-2H-pyrano[6,5-f]chromen-3-yl]-5-ı	RXRA
7-Acetoxy-2-methylisoflavone	RXRA
Vestitol	RXRA
Licoagrocarpin	RXRA
Odoratin	RXRA
shinpterocarpin	RXRB
Glabridin	RXRB
2-[(3R)-8,8-dimethyl-3,4-dihydro-2H-pyrano[6,5-f]chromen-3-yl]-5-ı	RXRB
Licoagrocarpin	RXRB
arachidonate	RXRG
quercetin	RUNX2
Glycyrol	CHEK1
Jaranol	CHEK1
isorhamnetin	CHEK1
Lupiwighteone	CHEK1
7-Methoxy-2-methyl isoflavone	CHEK1
formononetin	CHEK1
Calycosin	CHEK1
Glyasperin C	CHEK1
Isotrifoliol	CHEK1
(E)-1-(2,4-dihydroxyphenyl)-3-(2,2-dimethylchromen-6-yl)prop-2-en	CHEK1
kanzonols W	CHEK1
(2S)-6-(2,4-dihydroxyphenyl)-2-(2-hydroxypropan-2-yl)-4-methoxy-2	CHEK1
Semilicoisoflavone B	CHEK1
Glepidotin A	CHEK1
Phaseolinisoflavan	CHEK1
Glypallichalcone	CHEK1
Licochalcone B	CHEK1

3-(2,4-dihydroxyphenyl)-8-(1,1-dimethylprop-2-enyl)-7-hydroxy-5-m	CHEK1
Licoricone	CHEK1
Gancaonin A	CHEK1
Gancaonin B	CHEK1
3-(3,4-dihydroxyphenyl)-5,7-dihydroxy-8-(3-methylbut-2-enyl)chrom	CHEK1
5,7-dihydroxy-3-(4-methoxyphenyl)-8-(3-methylbut-2-enyl)chromone	CHEK1
2-(3,4-dihydroxyphenyl)-5,7-dihydroxy-6-(3-methylbut-2-enyl)chrom	CHEK1
Glycyrin	CHEK1
Licoisoflavone	CHEK1
Licoisoflavone B	CHEK1
Glyzaglabrin	CHEK1
Glabridin	CHEK1
Glabrone	CHEK1
1,3-dihydroxy-9-methoxy-6-benzofurano[3,2-c]chromenone	CHEK1
1,3-dihydroxy-8,9-dimethoxy-6-benzofurano[3,2-c]chromenone	CHEK1
Eurycarpin A	CHEK1
HMO	CHEK1
3'-Hydroxy-4'-O-Methylglabridin	CHEK1
licochalcone a	CHEK1
3'-Methoxyglabridin	CHEK1
2-[(3R)-8,8-dimethyl-3,4-dihydro-2H-pyrano[6,5-f]chromen-3-yl]-5-i	CHEK1
7,2',4'-trihydroxy－5-methoxy-3－arylcoumarin	CHEK1
7-Acetoxy-2-methylisoflavone	CHEK1
Vestitol	CHEK1
Gancaonin G	CHEK1
Licoagroisoflavone	CHEK1
Odoratin	CHEK1
Phaseol	CHEK1
dehydroglyasperins C	CHEK1
quercetin	CHEK2
diosgenin	MTOR
kaempferol	PPP3CA
piperlonguminine	SAA1
beta-sitosterol	PON1
quercetin	PON1
kaempferol	STAT1
quercetin	STAT1
licochalcone a	STAT3
Stigmasterol	SCN5A
Kadsurenone	SCN5A
hancinone C	SCN5A
Diop	SCN5A
beta-sitosterol	SCN5A
Tetrahydroalstonine	SCN5A
quercetin	SCN5A
hederagenin	SCN5A
Inermin	SCN5A
Frutinone A	SCN5A
Girinimbin	SCN5A
suchilactone	SCN5A
Fumarine	SCN5A
3β-acetoxyatractylone	SCN5A
Inermine	SCN5A
Jaranol	SCN5A
Medicarpin	SCN5A
Lupiwighteone	SCN5A
7-Methoxy-2-methyl isoflavone	SCN5A
euchrenone	SCN5A
glyasperin F	SCN5A
Glyasperin C	SCN5A
(E)-1-(2,4-dihydroxyphenyl)-3-(2,2-dimethylchromen-6-yl)prop-2-en	SCN5A
kanzonols W	SCN5A
Semilicoisoflavone B	SCN5A
Glepidotin A	SCN5A
Glepidotin B	SCN5A
Phaseolinisoflavan	SCN5A
Glypallichalcone	SCN5A
Gancaonin A	SCN5A
2-(3,4-dihydroxyphenyl)-5,7-dihydroxy-6-(3-methylbut-2-enyl)chrom	SCN5A
licoisoflavanone	SCN5A
shinpterocarpin	SCN5A
Glabridin	SCN5A
Glabranin	SCN5A

Glabrene	SCN5A
Glabrone	SCN5A
Eurycarpin A	SCN5A
(2S)-7-hydroxy-2-(4-hydroxyphenyl)-8-(3-methylbut-2-enyl)chroman	SCN5A
HMO	SCN5A
1-Methoxyphaseollidin	SCN5A
Quercetin der.	SCN5A
3'-Hydroxy-4'-O-Methylglabridin	SCN5A
licochalcone a	SCN5A
3'-Methoxyglabridin	SCN5A
2-[(3R)-8,8-dimethyl-3,4-dihydro-2H-pyrano[6,5-f]chromen-3-yl]-5-[illegible]	SCN5A
Inflacoumarin A	SCN5A
6-prenylated eriodictyol	SCN5A
7-Acetoxy-2-methylisoflavone	SCN5A
8-prenylated eriodictyol	SCN5A
Vestitol	SCN5A
Licoagrocarpin	SCN5A
Glyasperins M	SCN5A
Licoagroisoflavone	SCN5A
Odoratin	SCN5A
dehydroglyasperins C	SCN5A
Stigmasterol	SLC6A3
piperlonguminine	SLC6A3
Aposiopolamine	SLC6A3
Fumarine	SLC6A3
Medicarpin	SLC6A3
7-Methoxy-2-methyl isoflavone	SLC6A3
formononetin	SLC6A3
Glypallichalcone	SLC6A3
HMO	SLC6A3
licochalcone a	SLC6A3
2-[(3R)-8,8-dimethyl-3,4-dihydro-2H-pyrano[6,5-f]chromen-3-yl]-5-[illegible]	SLC6A3
Vestitol	SLC6A3
Stigmasterol	SLC6A2
kaempferol	SLC6A2
hederagenin	SLC6A2
Aposiopolamine	SLC6A2
piperlonguminine	SLC6A4
beta-sitosterol	SLC6A4
Tetrahydroalstonine	SLC6A4
Inermin	SLC6A4
Aposiopolamine	SLC6A4
Fumarine	SLC6A4
DFV	SLC6A4
Medicarpin	SLC6A4
7-Methoxy-2-methyl isoflavone	SLC6A4
formononetin	SLC6A4
Glypallichalcone	SLC6A4
(2R)-7-hydroxy-2-(4-hydroxyphenyl)chroman-4-one	SLC6A4
HMO	SLC6A4
Vestitol	SLC6A4
kaempferol	SLC2A4
quercetin	SLC2A4
ginsenoside rh2	SLC2A4
naringenin	SOAT1
naringenin	SOAT2
naringenin	SREBF1
quercetin	MMP3
diosgenin	SOD1
quercetin	SOD1
naringenin	SOD1
liquiritin	SOD1
Kadsurenone	F2
hancinone C	F2
Cornudentanone	F2
Tetrahydroalstonine	F2
kaempferol	F2
quercetin	F2
Frutinone A	F2
3β-acetoxyatractylone	F2
Glycyrol	F2
isorhamnetin	F2
Lupiwighteone	F2

7-Methoxy-2-methyl isoflavone	F2
formononetin	F2
glyasperin B	F2
Glyasperin C	F2
(2S)-6-(2,4-dihydroxyphenyl)-2-(2-hydroxypropan-2-yl)-4-methoxy-2	F2
Semilicoisoflavone B	F2
Glepidotin A	F2
3-(2,4-dihydroxyphenyl)-8-(1,1-dimethylprop-2-enyl)-7-hydroxy-5-m	F2
Licoricone	F2
Gancaonin A	F2
Gancaonin B	F2
3-(3,4-dihydroxyphenyl)-5,7-dihydroxy-8-(3-methylbut-2-enyl)chrom	F2
2-(3,4-dihydroxyphenyl)-5,7-dihydroxy-6-(3-methylbut-2-enyl)chrom	F2
Glycyrin	F2
Licoisoflavone	F2
Licoisoflavone B	F2
licopyranocoumarin	F2
Glabrone	F2
Eurycarpin A	F2
Isolicoflavonol	F2
1-Methoxyphaseollidin	F2
Inflacoumarin A	F2
7-Acetoxy-2-methylisoflavone	F2
Gancaonin G	F2
Licoagrocarpin	F2
Licoagroisoflavone	F2
Phaseol	F2
quercetin	THBD
quercetin	PLAT
beta-sitosterol	JUN
kaempferol	JUN
quercetin	JUN
formononetin	JUN
quercetin	E2F1
quercetin	E2F2
diosgenin	RELA
kaempferol	RELA
quercetin	RELA
isorhamnetin	RELA
naringenin	RELA
licochalcone a	RELA
beta-sitosterol	TGFB1
quercetin	TGFB1
Kadsurenone	PRSS1
hancinone C	PRSS1
Hydroxygenkwanin	PRSS1
Tetrahydroalstonine	PRSS1
kaempferol	PRSS1
quercetin	PRSS1
Inermin	PRSS1
Inermine	PRSS1
Jaranol	PRSS1
Medicarpin	PRSS1
isorhamnetin	PRSS1
Lupiwighteone	PRSS1
7-Methoxy-2-methyl isoflavone	PRSS1
formononetin	PRSS1
Calycosin	PRSS1
glyasperin B	PRSS1
glyasperin F	PRSS1
Glyasperin C	PRSS1
kanzonols W	PRSS1
(2S)-6-(2,4-dihydroxyphenyl)-2-(2-hydroxypropan-2-yl)-4-methoxy-2	PRSS1
Semilicoisoflavone B	PRSS1
Glepidotin A	PRSS1
Phaseolinisoflavan	PRSS1
3-(2,4-dihydroxyphenyl)-8-(1,1-dimethylprop-2-enyl)-7-hydroxy-5-m	PRSS1
Licoricone	PRSS1
Gancaonin A	PRSS1
Gancaonin B	PRSS1
3-(3,4-dihydroxyphenyl)-5,7-dihydroxy-8-(3-methylbut-2-enyl)chrom	PRSS1
5,7-dihydroxy-3-(4-methoxyphenyl)-8-(3-methylbut-2-enyl)chromone	PRSS1
2-(3,4-dihydroxyphenyl)-5,7-dihydroxy-6-(3-methylbut-2-enyl)chrom	PRSS1

Glycyrin	PRSS1
Licoisoflavone	PRSS1
Licoisoflavone B	PRSS1
licoisoflavanone	PRSS1
shinpterocarpin	PRSS1
licopyranocoumarin	PRSS1
Glyzaglabrin	PRSS1
Glabridin	PRSS1
Glabrene	PRSS1
Glabrone	PRSS1
Eurycarpin A	PRSS1
Isolicoflavonol	PRSS1
HMO	PRSS1
1-Methoxyphaseollidin	PRSS1
Quercetin der.	PRSS1
3'-Hydroxy-4'-O-Methylglabridin	PRSS1
3'-Methoxyglabridin	PRSS1
2-[(3R)-8,8-dimethyl-3,4-dihydro-2H-pyrano[6,5-f]chromen-3-yl]-5-r	PRSS1
Inflacoumarin A	PRSS1
7-Acetoxy-2-methylisoflavone	PRSS1
Vestitol	PRSS1
Gancaonin G	PRSS1
Gancaonin H	PRSS1
Licoagrocarpin	PRSS1
Glyasperins M	PRSS1
Glycyrrhiza flavonol A	PRSS1
Licoagroisoflavone	PRSS1
Odoratin	PRSS1
dehydroglyasperins C	PRSS1
kaempferol	TNF
quercetin	TNF
ginsenoside rh2	TNF
kaempferol	DIO1
quercetin	DIO1
naringenin	UGT1A1
Stigmasterol	PLAU
quercetin	PLAU
kaempferol	VCAM1
quercetin	VCAM1
diosgenin	VEGFA
quercetin	VEGFA
Fumarine	KDR
Glycyrol	KDR
glyasperin B	KDR
(2S)-6-(2,4-dihydroxyphenyl)-2-(2-hydroxypropan-2-yl)-4-methoxy-2	KDR
Glepidotin A	KDR
licochalcone G	KDR
3-(2,4-dihydroxyphenyl)-8-(1,1-dimethylprop-2-enyl)-7-hydroxy-5-m	KDR
Licoricone	KDR
Gancaonin B	KDR
Glycyrin	KDR
Licoisoflavone	KDR
liquiritin	KDR
licopyranocoumarin	KDR
Sigmoidin-B	KDR
1-Methoxyphaseollidin	KDR
3'-Hydroxy-4'-O-Methylglabridin	KDR
Gancaonin H	KDR
Glyasperins M	KDR
Phaseol	KDR
Fumarine	CACNA1S
kaempferol	XDH
quercetin	XDH
isorhamnetin	XDH
